# Characterization of novel low passage primary and metastatic colorectal cancer cell lines

**DOI:** 10.18632/oncotarget.7391

**Published:** 2016-02-15

**Authors:** Arnoud Boot, Jaap van Eendenburg, Stijn Crobach, Dina Ruano, Frank Speetjens, Jan Calame, Jan Oosting, Hans Morreau, Tom van Wezel

**Affiliations:** ^1^ Department of Pathology, Leiden University Medical Center, Leiden, The Netherlands; ^2^ Department of Clinical Oncology, Leiden University Medical Center, Leiden, The Netherlands

**Keywords:** colorectal cancer cell line, chemosensitivity, next generation sequencing, copy number profiling, gene expression

## Abstract

*In vitro* models are essential to understanding the molecular characteristics of colorectal cancer (CRC) and the testing of therapies for CRC. Many efforts to establish and characterize primary CRC cell lines have been published, most describing a small number of novel cell lines. However, there remains a lack of a large panel of uniformly established and characterized cell lines. To this end we established 20 novel CRC cell lines, of which six were derived from liver metastases. Genetic, genomic and transcriptomic profiling was performed in order to characterize these new cell lines. All data are made publically available upon publication.

By combining mutation profiles with CNA and gene expression profiles, we generated an overall profile of the alterations in the major CRC-related signaling pathways. The combination of mutation profiles with genome, transcriptome and methylome data means that these low passage cell lines are among the best characterized of all CRC cell lines. This will allow researchers to select model cell lines appropriate to specific experiments, facilitating the optimal use of these cell lines as *in vitro* models for CRC. All cell lines are available for further research.

## INTRODUCTION

With over a million cases diagnosed every year, colorectal cancer (CRC) is the third most commonly occurring cancer in the world (Globocan 2012). The occurrence of metastatic disease has a major impact on patient survival: CRC patients presenting with distant metastases have a 5-year survival rate of only 12%, while patients with local disease or regional spreading of disease show 5-year survival rates of 90% and 70%, respectively [1].

One third of CRC cases already show distant metastases at diagnosis and around 50-60% of these metastases are found in the liver [2–4]. Metastatic CRC (mCRC) is often treated using the FOLFOX protocol, a series of chemotherapy treatments consisting of oxaliplatin and 5-FU. mCRC without *RAS* mutations are treated with EGFR inhibition therapies such as cetuximab and panitumumab. Patients without *RAS* mutations show a median overall survival of 23.8 months on this treatment [5]. Patients with *RAS* mutant CRC are ineligible for EGFR inhibition therapy and show a median overall survival of 19.2 months.

Development of new drugs for the treatment of cancer starts with *in vitro* testing of candidate compounds. The availability of a cell line model that closely resembles the tumor subtype under investigation is therefore pivotal. Many well characterized cell lines exist which represent most of the CRC subtypes [6, 7]. These CRC cell lines, such as HCT116, HT-29, SW480 and LoVo, were established several decades ago [8–11]. For many of these cell lines clinicopathological parameters and information on patient characteristics are incomplete. More importantly, these cell lines have been in culture for decades and have likely diverged from initial cultures at both the genetic and epigenetic levels. This creates the concern that these cell lines might be less suitable for pharmacological testing as representative CRC models [12, 13]. Lange et al. for example state: “In contrast to cell lines of high passage, low-passage cancer cell lines well reflect the biology of the original tumor, such as growth behavior, morphology, and mutational profile and are, therefore, in our experience, a versatile tool to evaluate drug efficiencies in a preclinical context”, emphasizing that low passage cell lines are pivotal for pre-clinical drug screening [12].

Several studies were performed to establish and characterize low passage CRC cell lines. For example Maletzki et al. established CRC cell lines from 5 tumors, and extensively characterized their morphology, growth kinetics, and molecular profile [14, 15]. Several other publications describe the characterization of single low passage CRC cell lines [13, 16–18]. While an important contributions to the field, these studies all focus on various different aspects of cell line characterization and chemosensitivity. However, a uniform and comprehensive molecular characterization of low passage CRC cell lines is lacking.

For this reason we sought to generate novel CRC cell models and have now established a panel of 20 new CRC cell lines. Six of these originated from CRC liver metastases, while the remaining cell lines all derived from primary CRC tumors. We performed characterization of these novel CRC cell lines, including somatic mutation profiling genomic and transcriptomic analyses. Additionally, sensitivity to oxaliplatin was tested as a measure of sensitivity to current CRC treatment regimens. The combined dataset are publically available. These novel CRC cell lines will serve as a valuable research tool in addition to currently available cell lines to be used for *in vitro* drug research and may help further understanding of the molecular mechanisms underlying CRC.

## RESULTS

Here we report the establishment of 20 novel CRC cell lines, 14 of which were derived from primary colorectal cancers, while the remaining 6 were established from liver metastases. To assure these cell lines are permanent and stable, the cell lines were cultured for at least 30 passages. For the analyses described here cultures of approximately 13 passages were used. All cell lines have successfully been cultured multiple times from frozen vials to ensure they are able to survive the freezing process. The recovery rate was between 60-90%, except for JVE774 (10%).

We performed comprehensive genomic profiling of the cell lines, including genome-wide gene expression, copy number and somatic mutation analyses.

### Clinicopathological characteristics

Of the cell lines derived from primary tumors, five originated from distal CRC, including rectal and sigmoid tumors, while 9 originated from proximal CRC tumors, amongst others from the cecum and the ascending colon. The various tumor locations are listed in Table 1. Six of the 20 cell lines were derived from CRC liver metastases. Histological classification of the tumors from which these cell lines were derived were extracted from the pathology report. The majority of primary tumors were colorectal adenocarcinomas, including 4 mucinous adenocarcinomas.

**Table 1:** Cell line characteristics and mutation profiles

Cell line	Age	Gender	Location	Tumor morpho-logy	Dukes stage	Cell line growth	MSI	*BRAF*	*KRAS*	*PIK3CA*	*TP53*	*APC*	*SMAD4*	Other	*MLH1*	*MGMT*	*CDKN2A*
JVE015	66	F	S	--	--	Monolayer	MSS	--	c.34G>T	c.3140A>G	c.916C>T	c.3340C>T	--	--	U	M	M
JVE044	83	F	R	--	--	Piled-up	MSS	--	c.35G>T	--	c.524G>A	--	c.931C>T	*FBXW7*: c.1513C>T	U	U	M
JVE059	58	M	T	AC	D	Monolayer	MSI-H	--	--	--	--	--	--	*EGFR*: c.2164G>A *MLH1*: c.551C>A *MLH1*: c.1975C>T	U	U	P
JVE103	51	M	Meta	AC	D	Piled-up	MSS	--	--	--	--	--	--	*SMAD3*: HomDel	U	U	U
JVE109	84	F	H	AC	C1	Monolayer	MSI-H	c.1799T>A	--	--	c.842A>G	--	--	*VHL*: c.449A>G *FGFR2*: c.544G>A	M	U	M
JVE114	71	M	Meta	AC	D	Monolayer	MSS	--	--	--	c.638G>T	c.3921_3925del	--	--	U	U	U
JVE127	60	M	A	MAC	C2	Monolayer	MSS	c.1799T>A	--	--	c.394A>C	--	--	*PTEN*: HomDel	U	U	U
JVE187	60	F	Meta	AC	D	Monolayer	MSS	--	c.351A>T	c.1636C>A	--	c.4668_4669del	c.1082G>A	*ERBB2*: c.2524G>A	U	M	M
JVE192	43	F	A	MAC	C1	Monolayer	MSI-H	--	c.38G>A	c.3062A>G	c.743G>A	--	--	*MLH1*: c.112A>C *FGFR3*: c.1906G>A *CTNNB1*: c.121A>G *GNAS*: c.601C>T	U	U	P
JVE207	67	M	D	AC	C2	Monolayer	MSS	c.1799T>A	--	--	c.527G>A	--	HomDel	--	U	U	U
JVE222	67	F	A	AC	B2	Monolayer	MSI-H	--	c.38G>A	c.263G>A	--	c.4348C>T	--	*MSH6*: c.2718_ 2719del	U	U	P
JVE241	79	M	C	MAC	B2	Piled-up	MSS	--	c.35G>T	--	c.257_279del	c.4496G>T	--	--	U	U	U
JVE253	48	F	Meta	MAC	D	Monolayer	MSS	--	c.35G>T	--	c.742C>T	c.4709_4713del	--	--	U	U	P
JVE367	61	F	I	LNEC	C1	Suspension	MSS	c.1799T>A	--	--	--	--	--	--	U	U	M
JVE371	67	M	Meta	AC	D	Monolayer	MSS	--	c.34G>A	c.1633G>A	c.673-1G>A	--	--	--	U	M	U
JVE528	57	F	A	AC	B1	Piled-up	MSS	--	c.38G>A	--	c.1024C>T	c.4348C>T	--	--	U	M	P
JVE774	61	M	R	AC	B1	Monolayer	MSS	--	c.64C>A	--	--	c.4033G>T	--	--	U	P	P
KP283T	49	F	Meta	AC	D	Monolayer	MSS	--	c.34G>T	--	c.818G>A	--	--	*ALK*: c.3599C>T *PTEN*: HomDel	U	U	U
KP363T	80	M	Ls	AC	B2	Monolayer	MSS	c.1799T>A	--	c.1633G>A	c.916C>T	--	HomDel	--	U	U	M
KP7038T	34	M	Rs	AC	B1	Monolayer	MSI-H	--	--	--	--	c.3292_3293del	--	*MLH1*: exon16 HomDel	U	U	U

Abbreviations: A = Ascending colon, C = Cecum, D = Descending colon, H = Hepatic flexure, i = Ileocecal junction, Ls = Left-sided colon, Meta = Liver metastasis, R = Rectum, Rs = right-sided colon, S = sigmoid, T = Colon Transversum, AC = AdenoCarcinoma, MAC = Mucinous AdenoCarcinoma, LNEC = Largecell Neuro-Endocrine Carcinoma, U = unmethylated, M = methylated, P = Partially methylated

Line JVE367 was derived from a large-cell neuroendocrine carcinoma (LNEC). LNEC account for only 0.2% of all CRC, and generally are associated with a very poor prognosis. [19, 20] To our knowledge JVE367 is the first LNEC cell line of colonic origin and offers an in vitro model to study this aggressive CRC subtype.

### Cell line morphology and identity

**Figure 1:**
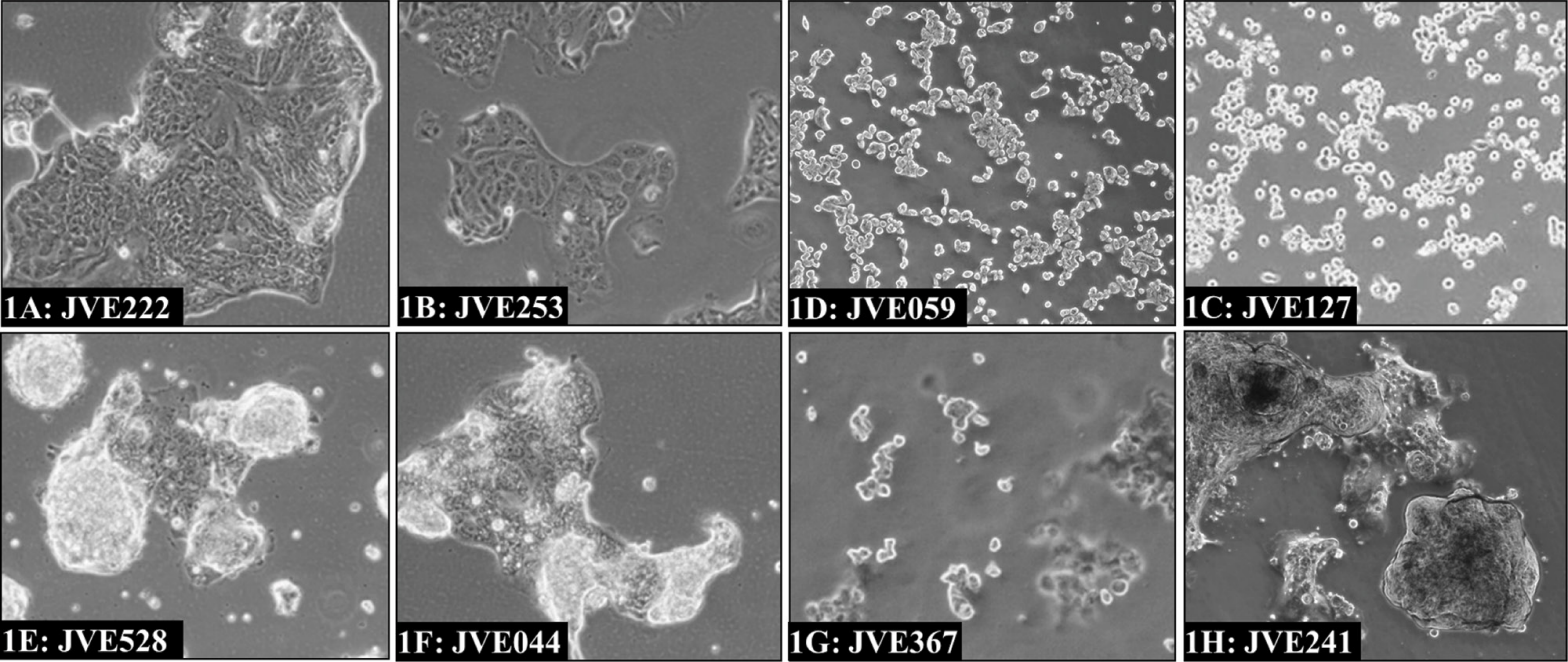
Examples of different cell line morphologies The predominant morphological growth patterns among the newly established cell lines include islands of cells in monolayers as illustrated by JVE222 and JVE253 **1A. and 1B.** a rounded off morphology as seen for JVE127 and JVE059 **1C. and 1D.** and cells with a piled-up morphology interspersed with monolayers, of which JVE528 **1E.** and JVE044 **1F.** are examples. JVE367 **1G.** is the only cell line that grows in suspension, while JVE241 **1H.** grows in a piled-up fashion but lacks the monolayers typical of the other cell lines with piled-up morphology.

Considerable differences in cell morphology could be observed between the cell lines. The various morphologies are illustrated in Figure 1 and growth characteristics are given in Table 1. Morphologically, two groups can be distinguished, with cells growing either in a monolayer or piled-up. Cells growing in monolayers consist of stretched cells, which grow as non-overlapping islands of cells (Figure 1A; JVE222 and 1B: JVE253). JVE059 and JVE127 also grow in monolayers but appear to be less adherent and form a layer of rounded, single cells (1C and 1D, respectively). The cell lines with a piled-up morphology form clumps of multi-layer cells, interspersed with cells in monolayer. Examples are shown in 1E (JVE528) and 1F (JVE044). Two cell lines, JVE367 and JVE241, show more unusual growth patterns. JVE367 (Figure 1G) grows in suspension, both in clumps and as single cells, and remains in suspension even when cells are transferred to collagen or gelatin-coated culture flasks. JVE241 (Figure 1H) shows a piled-up morphology, without cells that form a monolayer between the piled-up islands. Representative images of the other 12 cell lines are included in Supplementary Figure S1. Cell line identity was analyzed using short tandem repeat (STR) profiles (Supplementary Table S2). All cell lines are unique, and do not match any known cell line in the DSMZ database.

### Sensitivity to chemotherapeutics

**Figure 2:**
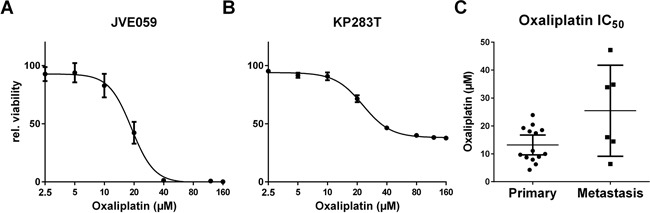
Oxaliplatin resistance Dose-response curves were generated for all cell lines in order to determine IC_50_ values. Examples are shown for JVE059 **A.** and KP283T **B.** with the latter showing high oxaliplatin resistance. **C.** shows the IC_50_ values grouped in either primary or metastasis-derived cell lines, and indicates an enrichment of resistant cell lines amongst the metastasis-derived cell lines (*p* < 0.02). Bars represent the mean, with 95% confidence intervals.

As a measure of resistance to chemotherapeutic regimens used in the treatment of CRC, sensitivity to oxaliplatin was assessed. Cell lines fell into one of three sensitivity groups; a resistant group (IC_50_ > 25 μM, N = 4), the high sensitivity group with IC_50_'s below 10 μM (N = 7) and an intermediate group with IC_50_'s between 10 μM and 25 μM (N = 9). Dose-response curves for JVE059 and KP283T are shown in Figure 2A and 2B as examples of sensitive and resistant cell lines. As shown in Figure 2C, cell lines derived from liver metastases showed a lower sensitivity to oxaliplatin (*t*-test, p < 0.02). IC_50_ values for oxaliplatin, including 95% confidence intervals, are included in Supplementary Table S1.

### DNA mismatch repair deficiency

While only 15% of primary CRC tumors show MSI-H [21] these cell lines are overrepresented in the available collections. In the Seltar database (www.seltarbase.org, accessed October 5^th^ 2015) 73 out of 147 CRC cell lines are MSI-H and recently Medico shows 41,7% of cell lines are MSI-H [7]. Five of our 20 cell lines (25%) were found to be MSI-H, all of which originate from tumors of the proximal colon. In sporadic CRC, MSI is most often caused by hypermethylation of the *MLH1* promoter, resulting in loss of the MutL complex. Therefore, *MLH1* DNA promoter hypermethylation was assessed. JVE109 was found to be hypermethylated at the *MLH1* promoter, while hypermethylation was absent in all other cell lines.

Amongst the most frequent targets in MSI-H tumors are the TGF-β and activin receptors, *TGFBR2* and *ACVR2A*. The microsatellites in exon 3 of *TGFBR2* and the 3^rd^ and 10^th^ exons *ACVR2A* were screened in all cell lines (Supplementary Table S3). Mutations in the *TGFBR2* microsatellite and in the 10^th^ exon of *ACVR2A* were found in four MSI-H cell lines. None of the cell lines showed instability of the microsatellite in exon 3 of *ACVR2A*, in concordance with previous results [21]. The fifth MSI-H cell line (JVE222) did not show instability at any of the *TGFBR2* and *ACVR2A* microsatellites.

### *CDKN2A* and *MGMT* promoter hypermethylation

In addition to *MLH1*, promoter hypermethylation of the *CDKN2A* and *MGMT* promoters was also assessed using methylation specific PCR (MSP). Hypermethylation of *MGMT* is observed in 38% [22] and *CDKN2A* methylation is found in 25% of CRCs [23]. In CRC cell lines, *CDKN2A* is methylated in up to 75% of cases [24, 25], and *CDKN2A* hypermethylation has been proposed to be part of a cell line-specific DNA methylation pattern [24]. MSP identified 5 cell lines that were hypermethylated for *MGMT*: of these only JVE774 showed partial methylation. MSP of *CDKN2A* identified 12 cell lines with hypermethylation of *CDKN2A*, of which 6 cell lines were partially methylated. Partial hypermethylation can be explained by either methylation of one of the alleles or by a mixed population of cells with and without hypermethylation at these loci. A more accurate quantification of the percentage of methylated alleles could shed more light on this.

### Somatic mutation profiling

To further characterize the cell lines, somatic mutations were studied in mutation hotspot regions of 50 known oncogenes and tumor suppressor genes, including *KRAS, NRAS, PIK3CA* and *BRAF* hotspots and part of the mutation cluster regions of *APC* and *TP53*. The most frequent mutated gene is *TP53*, with damaging mutations found in 13 cell lines.

Activating mutations in *KRAS* were found in 11 cell lines, including the less common *KRAS* activating mutations c.64C>A (p.Q22K) and c.351A>T (p.K117N). *APC* mutations were found in 9 cell lines.

We identified 5 cell lines with a *BRAF* c.1799T>A (p.V600E). Notably, all *BRAF*-mutated cell lines derived from primary CRC, while *KRAS* mutations were found in both primary and metastasis-derived cell lines. We identified *PIK3CA* mutations in 6 cell lines and all these cell lines also carried either a *KRAS* or a *BRAF* mutation. Co-occurrence of *PIK3CA* mutations with activating mutations in the mitogen activated protein kinase (MAPK) pathway was also observed in 74% of the TCGA colon adenocarcinoma samples. Damaging *SMAD4* mutations were found in 2 cell lines. Additionally, next generation sequencing (NGS) data revealed homozygous deletions of *SMAD4* in two additional cell lines. Similarly, *PTEN* homozygous deletions were observed in KP283T and JVE127, although no *PTEN* mutations were found. A possible activating *EGFR* c.2164G>A mutation as found in JVE059, however, the effect of this mutation is currently unknown. In the 4 MSI-H cell lines without *MLH1* methylation, NGS of the DNA mismatch repair genes *MLH1*, *MSH2*, *MSH6* and *PMS2* was performed. *MLH1* mutations were identified in JVE192 and JVE059. JVE192 carried the c.112A>C, p.N38H mutation, while JVE059 carried the compound heterozygous nonsense mutations c.551C>A and c.1975C>T. A homozygous *MSH6* mutation, c.2718_2719del, p.V907Rfs*10, was found in JVE222. Analysis of KP7038T failed to show a variant, but did reveal lack of coverage of exon 16 of *MLH1*, suggesting the presence of a homozygous deletion. We carried out PCR analysis spanning exons 15-17 of *MLH1* in KP7038T. The expected PCR product of 6497 bp was absent and was replaced by a PCR product of approximately 1350 bp. Sequencing analysis confirmed a deletion of 5146 bp at g.chr3:37084101-37089246, covering *MLH1* exon 16 and resulting in an in-frame deletion of 55 amino acids. This deletion disrupts the *PMS2* interaction domain, explaining the MSI-H profile of this cell line. An overview of all mutations, deletions and the promoter hypermethylation identified is provided in Table 1. The corresponding protein alterations resulting from these mutations are listed in Supplementary Table S5.

### Genome and transcriptome analysis

Complementary to the mutation profiles we generated genomic and transcriptomic profiles of all cell lines by hybridizing DNA and RNA respectively to Infinium HumanExome-12v1 BeadChips. Log2 relative gene expression values are included in Supplementary File 1.

### Gene expression of *MLH1*, *PTEN* and *SMAD4*

We evaluated the gene expression at the loci with homozygous deletions in *SMAD4* and *PTEN*, and for promoter DNA hypermethylation of *MLH1*. *MLH1* promoter hypermethylation was detected in JVE109. Expression of *MLH1* was found to be 15-fold decreased in this sample compared to all other cell lines (Supplementary Figure S2).

The gene expression data for the samples with homozygous deletions in *SMAD4*, *PTEN* and *MLH1* do not show a complete loss of gene expression for these genes. This is due to our selection of SNPs from the gene expression dataset, which are homozygous in the DNA in all samples. In the samples with a homozygous deletion, the SNP's at these sites are not called due to low signal intensity. Therefore the gene expression values reported are only based on the SNP's which are not in the deleted area's. As a result of this, *PTEN* expression is not included in our gene expression dataset, as the array contains only one exonic *PTEN* probe, which is not called homozygous in the samples with a deletion. *PTEN* gene expression values were extracted manually from the raw data, and plotted in Supplementary Figure S2, as well as *SMAD4* expression levels.

**Figure 3:**
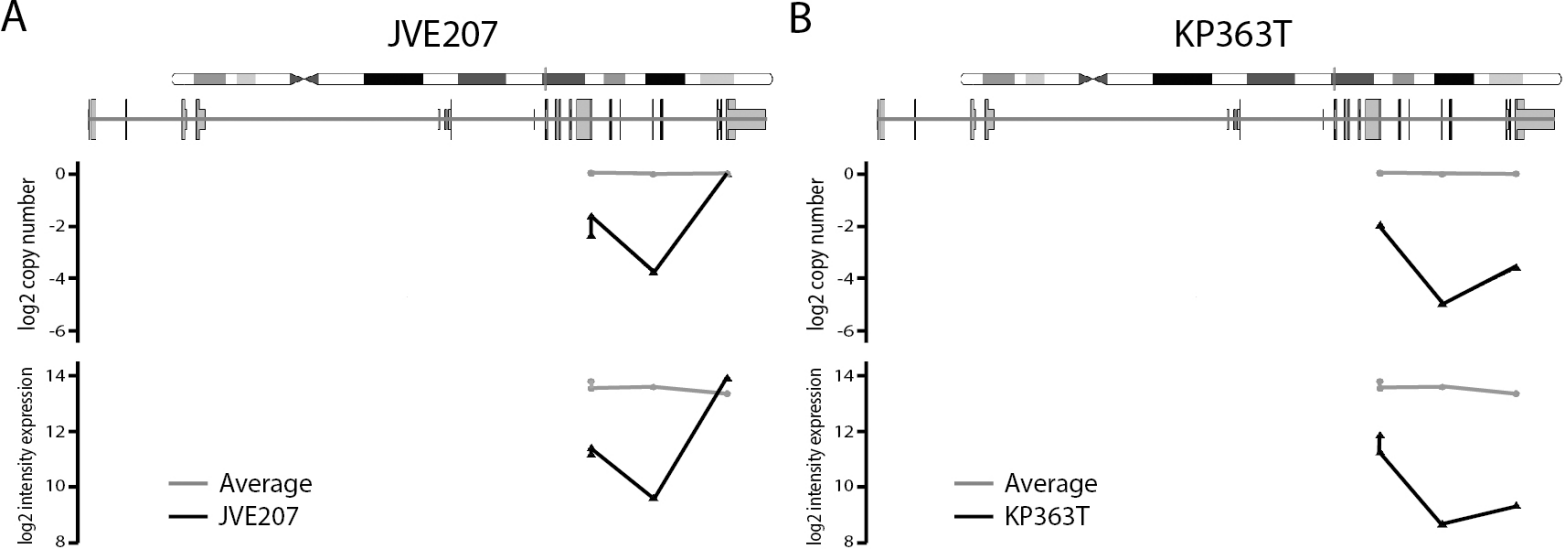
Probe level copy number and expression intensity for *SMAD4* in JVE207 and KP363T For both cell lines the copy number level and intensity in the expression data is compared to the average of all 20 cell lines. The JVE207 **A.** and KP363 **B.** data is plotted in black and the average of all 20 cell lines is shown in grey. Probes are plotted on their location in the *SMAD4* gene. All probes with reduced signal in the copy number data also show a reduction in intensity in the expression data.

To clarify this we plotted the average intensity in the DNA and expression datasets per probe for JVE207 (Figure 3A) and KP363T (Figure 3B), with the average intensity of all 20 cell lines in grey. Above the plot a schematic depiction of *SMAD4* is given to show the location in the gene. Both samples show a clear reduction in intensity in the expression data at all loci that show a copy number loss.

### Alterations in CRC signaling pathways

**Figure 5:**
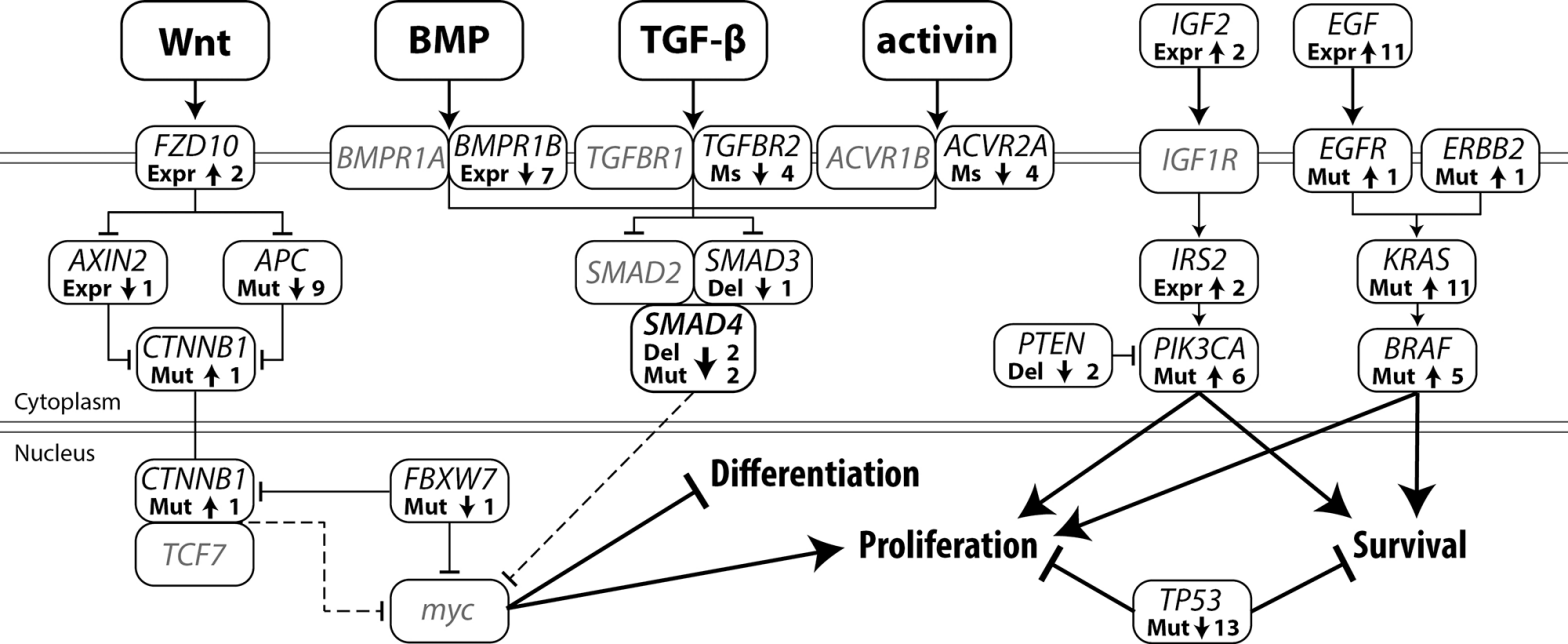
Somatic alterations in major CRC signaling pathways leading to a decrease in differentiation and increased proliferation and cell survival Arrow up: activating alteration, Arrow down: inactivating alteration, Expr: gene expression, Ms: microsatellite mutation, Mut: mutation, Del: deletion, Line with arrow: protein activation, Line with T-arrow: protein inhibition, Dotted line with T-arrow: inhibition of gene expression. The number represents the total number of cell lines with that specific alteration.

By combining the somatic mutation profiles and gene expression data, we were able to map alterations in the main signaling pathways. A graphic display of modifications in the Wnt, BMP/TGF-β, PI3K and receptor tyrosine kinase (RTK) pathways is given in Figure 5. As our expression dataset does not contain a reference, we depict cell lines which deviate from the majority as being altered. Most cell lines showed alterations in 2 or 3 of the signaling pathways, while JVE187, JVE192 and KP363T were altered for all 4 pathways. JVE114, a liver metastasis-derived cell line, was altered for only one of the pathways, indicating that tumors with few alterations in these pathways are also capable of developing metastases. Supplementary Table S3 shows all somatic alterations and gene expression values incorporated in this overview.

Upregulation of the MAPK signaling pathway is a common alteration in CRC and is generally mediated by activating mutations in *KRAS*, *BRAF* or *NRAS*. Alternatively, by upregulating *EGF* gene expression, cancer cells establish an autocrine feedback loop, abolishing their dependency on systemic EGF. Upregulation of *EGF* gene expression in CRC occurs in 9.7% of tumors (using a cutoff of 3x the median for normal colon samples) [26]. Upregulation of *EGF* was detected in 11 cases, 9 of which also showed a *KRAS* or *BRAF* mutation, and could therefore be considered to be no longer dependent on systemic EGF. JVE059 and JVE114 are the only cell lines that show neither a mutation in *BRAF* or *KRAS*, nor *EGF* upregulation. JVE059 harbors an *EGFR* mutation (c.2164G>A, p.A722T) within the tyrosine kinase domain, although it is not known whether this mutation leads to constitutive activation.

A *KRAS* c.351A>T, p.K117N mutation was identified in JVE187. This mutation is known to result in constitutive activation of MAPK signaling, albeit to a lower extent than mutations in codons 12, 13 and 22 [27]. Interestingly, the cell line also carries an *ERBB2* mutation, c.2524G>A, which is also known to result in constitutive activation of the MAPK pathway [28].

As a result of activating mutations in *PIK3CA* and inactivation of *PTEN*, PI3K signaling was activated in half of the cell lines. Alternative routes of activation for this pathway include transcriptional upregulation of *IGF2* and *IRS2*. JVE103 and JVE371 both showed a 4-fold increase in *IGF2* expression levels compared to the other samples. *IRS2* is known to be upregulated in a small proportion of CRC tumors [29]. Our dataset showed high *IRS2* expression levels in almost all cell lines, although it is difficult to draw any conclusions from this without reference values. JVE371 showed substantially lower *IRS2* expression levels, while it was amongs the highest *IGF2* expressing cell lines. As JVE371 also carries an activating *PIK3CA* mutation, it is probably independent of upstream signaling. JVE103, on the other hand, is wildtype for both *PIK3CA* and *PTEN*, which suggests that this cell line may have upregulated the PI3K pathway through an autocrine upregulation of *IGF2*.

### Metastasis-specific copy number alterations

DNA copy number profiles of each of the cell lines were generated using the Infinium HumanExome-12v1 data. In addition to copy number profiles, LOH and genomic imbalances were visualized by lesser allele intensity ratio (LAIR) analysis using the HumanExome-12v1 data [30]. Cell line copy number profiles and LAIR plots are included in Supplementary File 2.

**Figure 4:**
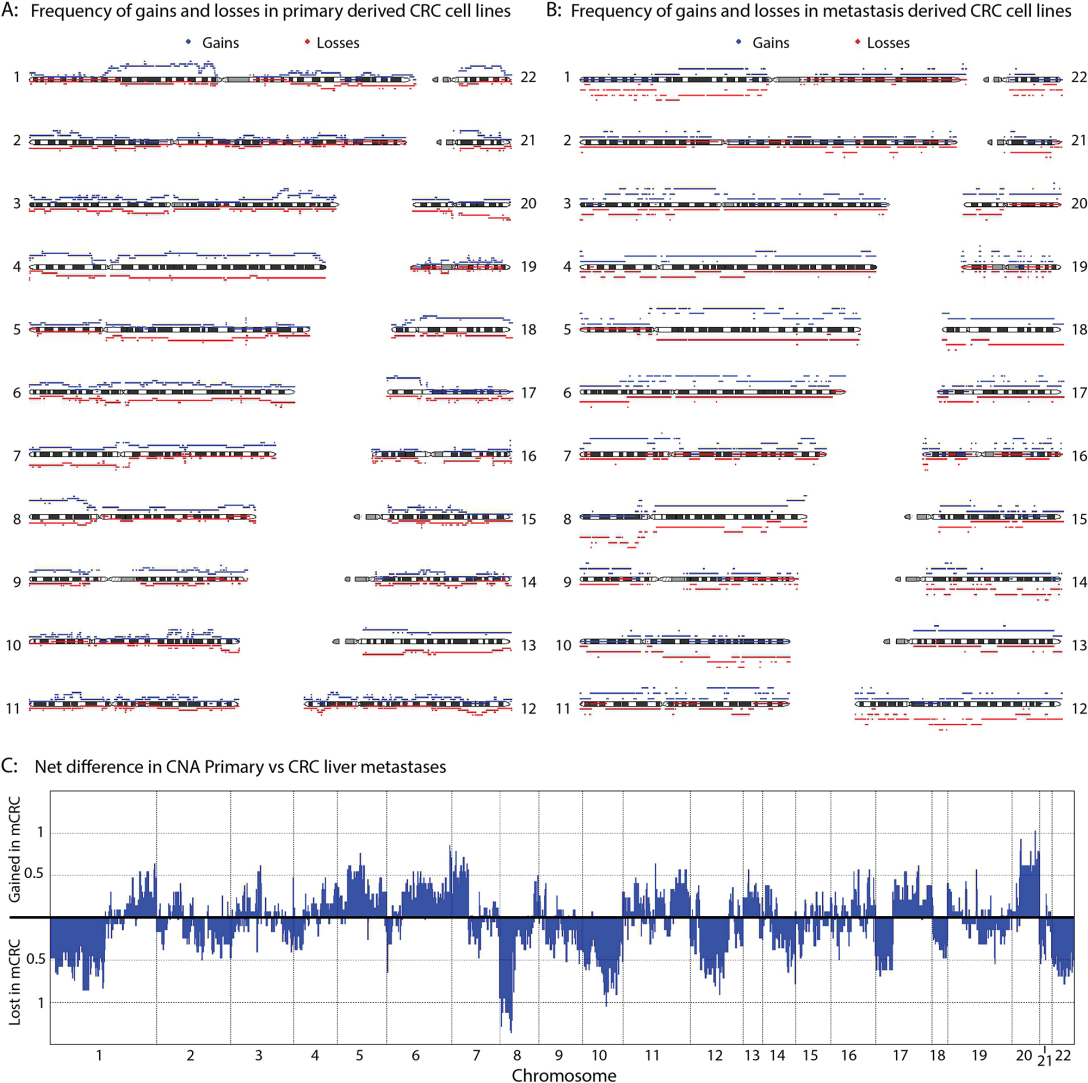
CNA comparison between primary and metastasis-derived cell lines The frequency of gains (red) and losses (blue) for the primary CRC derived cell lines **A.** and the liver metastasis cell lines **B.** is plotted. **C.** Combining the frequency of gains and losses for each position per group, total frequencies of gains and losses in the metastasis-derived cell lines compared to the primary CRC derived cell lines was calculated.

Differences in copy number profiles between CRC metastases and primary CRC tumors have been reported previously [31]. Liver metastases and tumors that later formed liver metastases reportedly show gains of chr20q. We therefore compared the copy number profiles of the CRC liver metastasis cell lines with those of the primary CRC tumors to determine whether this alteration could also be detected in these cell lines. The frequency of gains and losses per group are shown in Figure 4A (primary CRC derived cell lines) and Figure 4B (liver metastasis derived cell lines). We found chr20q amplification in 5 of the metastasis cell lines. In addition, 50% of the primary tumor derived cell lines showed a loss of chr20. This is further evidence that chr20q amplification is highly specific for liver metastases. A frequent gain of chr20q was also found in the TCGA data [32], and the comparative study of 63 cell lines [6].

To visualize the differences in copy number alterations (CNA) between primary tumor cell lines and metastasis-derived cell lines, the overall differences in CNA between the groups were plotted (Figure 4C). In addition to chr20q alterations, other chromosomes also show a clear difference between groups. For example, chromosomes 10 and 12 are lost predominantly in metastases, whereas chr11 is preferentially gained. Similar to the observations made concerning chr20q; chr7p was found to be lost in 40% of the primary cell lines, whereas 50% of the metastasis cell lines showed a gain of chr7q. The most marked difference between the metastases and primary tumor cell lines was CNA on chr8p. All of the liver metastasis cell lines showed a loss at chr8p, whereas around 70% of the primary cell lines showed a gain. Also both the TCGA data and a recent study of 63 cell lines showed a very high frequency of chr8p loss, similar to what we see in the metastasis cell lines[6]. The presence of metastasis-specific copy number alterations at these chromosomal locations could indicate that these regions harbor genes important for metastases.

## DISCUSSION

We established 20 novel cell lines from primary and metastasized CRC tumors. Using NGS and microarray-based approaches, we generated genetic, genomic and transcriptomic profiles and evaluated cell line sensitivity to oxaliplatin as a measure for chemoresistance. Thus we established a set of low passage CRC cell lines, which have all been thoroughly characterized in a uniform manner.

The characterization of these low passage cell lines identified a large diversity in mutation spectra and gene expression profiles. This extensive panel of new CRC cell lines represents a valuable research tool that can now be applied to *in vitro* experiments to further untangle the complexity of CRC tumorigenesis, and to the development of new therapies for both primary and metastatic disease.

The combined dataset presented here elevates these cell lines to rank among the best characterized CRC cell lines. The SNP array data will also be made available through GEO (accession numbers GSE67773 and GSE67774), allowing researchers to select appropriate cell line models for their particular experiment, thus allowing optimal use of these novel cell lines as *in vitro* models for CRC. The cell lines characterized in this manuscript are deposited at the Leibniz Institute DSMZ-German Collection of Microorganisms and Cell Cultures (www.dsmz.de).

## MATERIALS AND METHODS

### Cell line establishment and culture

Anonymized tumor material was thoroughly rinsed using RPMI-1640 medium under vigorous tapping. The tissue was cut into ± 1 mm^3^ fragments which were enzymatically dissociated using a 1% collagenase I-A (Sigma), 1% dispase (Gibco Life Technologies) solution. The dissociated cells were washed with RPMI-1640 and culturing was commenced in DMEM/F12 supplemented with 10% fetal bovine serum (FBS), 100 U penicillin and 100 μg streptomycin per mL. Once the cell line was established, further culturing was performed in either RPMI-1640 or DMEM/F12 medium (lines JVE222 and JVE241) supplemented with 10% FBS, 2 mM Glutamax-I, 50 U penicillin and 50 μg streptomycin per mL (all from Life Technologies, Grand Island, NY, USA). Cells were cultured at 37°C with 9% CO_2_ in a humidified stove. Anonymized samples were handled according to the medical ethical guidelines described in the Code Proper Secondary Use of Human Tissue established by the Dutch Federation of Medical Sciences. All cell lines were checked for mycoplasma using a mycoplasma-specific PCR [33].

### DNA isolation

DNA isolation was performed using the Wizard Genomic DNA Purification Kit (Promega, Madison, WI, USA) according to the manufacturer's instructions.

### RNA isolation

RNA isolation was performed on cells in exponential growth phase, using TRIzol® Reagent (Life Technologies). DNAse treatment was performed in suspension using rDNAse (Macherey Nagel GmbH & Co. KG, Düren, Germany).

### Cell line authentication

Short tandem repeat (STR) profiles of the cell lines were established using the CellID system (Promega) according to the manufacturer's instructions. Fragment analysis was performed on an Applied Biosystems 3130 Genetic Analyser (Life Technologies). STR profiles for all cell lines are included in Supplementary Table S2.

### Toxicity profiling

Cells were seeded in 96-well plates at 10000 cells per well. Twenty-four hours after seeding, the medium was removed and fresh medium containing oxaliplatin (Xeloda, Fresenius Kabi Nederland B.V., Zeist, The Netherlands) was added. After 72 hours of incubation with the compound, viability was assessed using the PrestoBlue® assay (Life Technologies) according to the manufacturer's instructions. Toxicity profiling was performed in triplicate and reproduced in 2 independent experiments. IC_50_ concentration was determined using Graphpad Prism® software (version 5.01).

### Microsatellite analysis

The microsatellite instability (MSI) status of each of the cell lines was determined using the MSI analysis system (Version 1.2, Promega) according to the manufacturer's instructions. Fragment analysis was performed on an Applied Biosystems 3130 Genetic Analyser (Life Technologies). Samples with at least two out of five unstable mononucleotide markers were classified as MSI-H. *TGFBR2* and *ACVR2* microsatellite analysis was performed as described previously [21].

### Methylation specific PCR

Bisulfite conversion was performed using the EZ DNA methylation gold kit (Zymo research, Orange, California, USA) according to the manufacturer's instructions, using an input of 200 ng of DNA. Bisulfite-converted DNA was eluted in 15 μL MQ water.

*MLH1* and *MGMT* MSPs were performed according to protocols developed at the Pathology Department molecular diagnostics lab at the LUMC [34]. *MGMT* methylation-specific PCR (MSP) was performed using the same protocol, using the following primers: MGMT_Um_Fw: TTTGTGTTTTGATGTTTGTAGGTTTTTGT, MGMT_Um_Rev: AACTCCACACTCTTCCAAAAACAAAACA, MGMT_M_Fw: TTTCGACGTTCGTAGGTTTTCGC, MGMT_M_Rev: GCACTCTTCCGAAAACGAAACG.

For the *CDKN2A*, 1 μL of bisulfite converted DNA was used in combination with 1 pmol primers in a total volume of 10 μl, containing 1x IQ SYBR Green supermix (Biorad, Hercules, California, USA). The primers used were as follows: CDKN2A-M-Fw: TGTAAAACGACGGCCAGTTTATTAGAGGGTGGGG CGGATCGC, CDKN2A-M-Rev: CAGGAAACAGC TATGACCGACCCCGAACCGCGACCGTAA, CDKN2A-Um-Fw: TGTAAAACGACGGCCAGTTTATTAGAG GGTGGGGTGGATTGT, CDKN2A-Um-Rev: CAGGA AACAGCTATGACCCAACCCCAAACCACAACCA TAA. PCR protocol: 5′ at 95°C, 40 cycles of 15″ at 95°C, 30″ at 69°C and 30″ at 72°C. *CDKN2A* methylation status was determined by gel analysis of the PCR product and by melting curve analysis.

### Somatic mutation profiling

Genomic DNA (10 ng) from each sample was used to prepare barcoded libraries using IonXpress barcoded adapters (Life Technologies). Libraries were pooled to a final concentration of 15 ng/mL after quantification with a fluorometer (Qubit HS, Life Technologies), and emulsion PCR was performed using the Ion PGM Template OT2 200 kit on a OneTouch-2 instrument. Sequencing was performed on an Ion Torrent Personal Genome Machine, using 316v2 chips.

Somatic mutations were analyzed using the Ion AmpliSeq™ Cancer Hotspot Panel v2. A list of the target genes included in this panel, along with mutation frequencies in CRC reported by the TCGA, is included in Supplementary Table S4 [29]. The pathogenicity of non-synonymous variants was assessed using PolyPhen2, MutationTaster and MutationAssessor. Mutations predicted to be damaging by at least 2 of these tools were considered to be pathogenic. Frameshift and nonsense mutations were considered to be always damaging.

Mutations in codon 600 of *BRAF*, codons 12 and 13 of *KRAS* and codons 545 and 1047 of *PIK3CA* were validated using Taqman genotyping assays [35].

### Infinium HumanExome-12 v1 BeadChips

Infinium HumanExome-12 v1 BeadChips were used with an input of 200 ng DNA. Raw data and preprocessed intensities per probe are available via the Gene Expression Omnibus (GEO) under accession numbers GSE67773 and GSE67774. For gene expression analysis using the same platform, 500 ng of RNA was converted to cDNA using the DyNAmo™ cDNA Synthesis Kit (Thermo Scientific, Waltham, MA, USA). cDNA was then purified using the QIAquick PCR purification kit (Qiagen, Germantown, Maryland, USA) and eluted in 15 μL MQ water. Five μl of purified cDNA was used as input for the Infinium protocol.

### Copy number and LAIR analysis

Lesser allele intensity ratio (LAIR) analysis was performed as described previously [30]. Copy number profiles and group copy number analysis was performed using the DNAcopy package [36]. For grouped copy number differences, gains and losses were called using a threshold of 0.10 deviation from the median copy number, as applied by GISTIC2.0 [37].

### Gene expression analysis

Gene expression data was generated using the intensity data of the cDNA hybridization on the Infinium HumanExome-12 v1 BeadChips. Intronic probes and probes which were heterozygous in any sample were removed. Subsequently, intensity data related to the color of the genotyped allele was extracted for each probe. After quantile normalization using the Limma package [38], the average probe intensity per gene was calculated and gene expression was reported in log2 expression values per gene per sample. In total 17090 genes were assayed. Gene expression values are included in Supplementary File 1.

## SUPPLEMENTARY FIGURES AND TABLES
















