# Supplementary material for: Characterization of novel low passage primary and metastatic colorectal cancer cell lines
Source: Oncotarget. 2016 Feb 15;7(12):14499–509. doi: 10.18632/oncotarget.7391 (PMC4924731; doi:10.18632/oncotarget.7391)
Supplement: Supplementary file 1 [file oncotarget-07-14499-s001.pdf]

SUPPLEMENTARY FIGURES AND TABLES

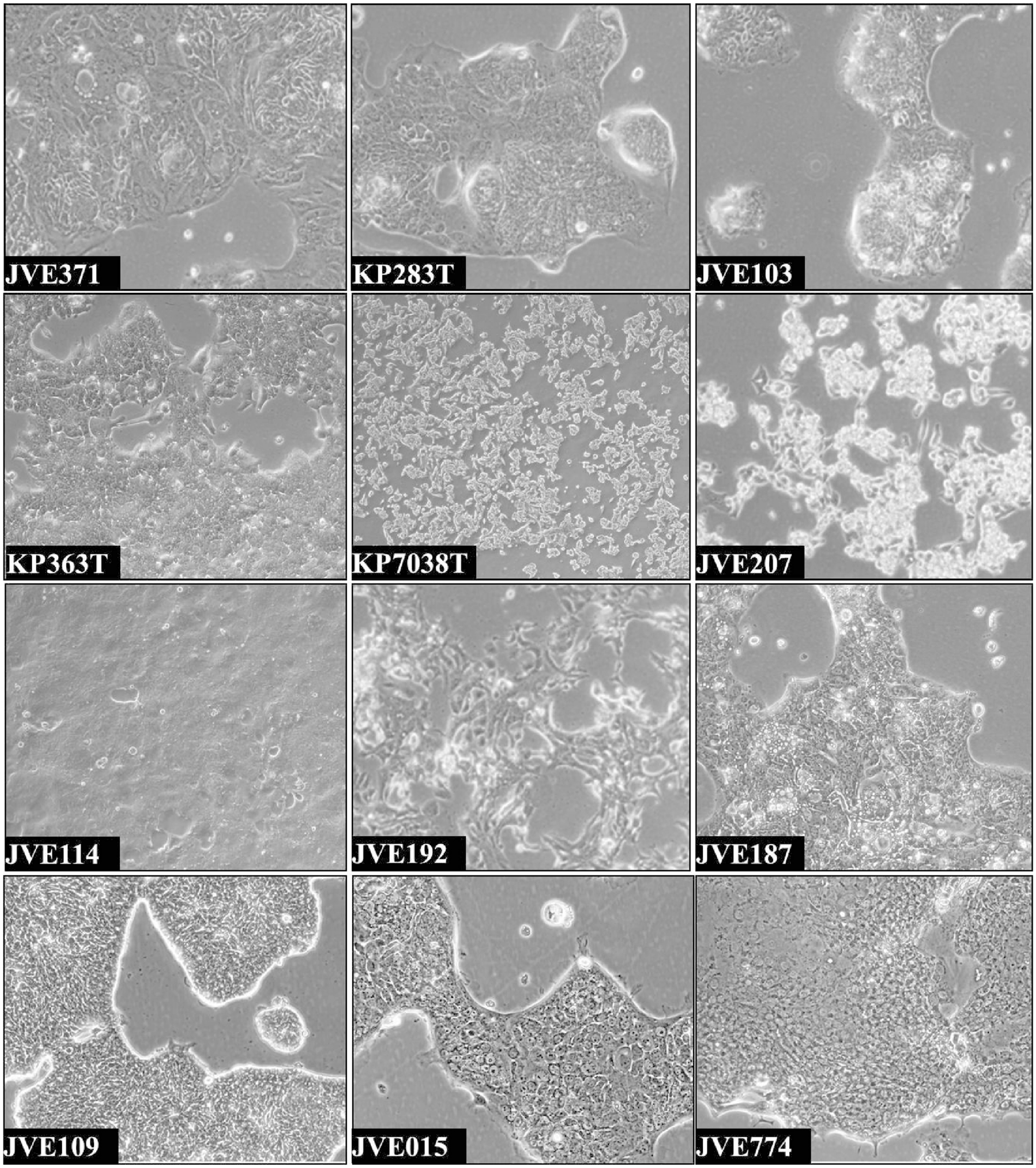

Supplementary Figure S1: Representative pictures for the remaining 12 cell lines, which were not included in Figure 1.

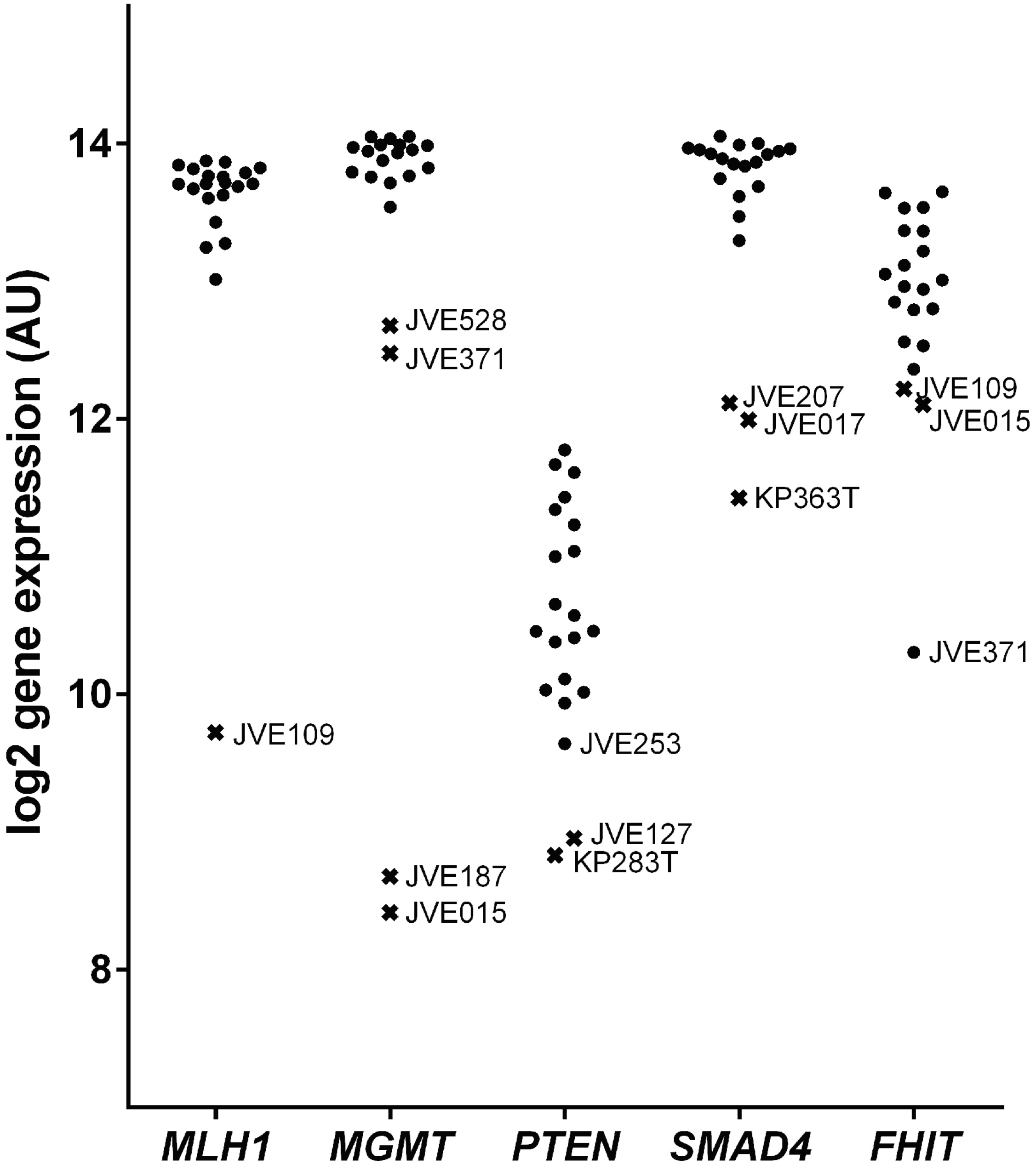

Supplementary Figure S2: Gene expression. Gene expression values of *MLH1*, *MGMT*, *PTEN*, *SMAD4* and *FHIT* are shown to show the effect of DNA methylation and homozygous deletions on gene expression. Cell lines with alterations (methylation or deletion) have been labelled. Gene expression of *MLH1* and *MGMT* are reduced by the presence of DNA methylation at the respective promoters. Homozygous deletion of *SMAD4* does not appear to result in a complete loss of expression. This is due to our expression analysis, selecting for probes outside of the deleted areas, as discussed in the main text.

Supplementary Table S1: Oxaliplatin IC50 with 95% confidence interval.
See Supplementary File 3

Supplementary Table S2: Cell line STR profiles as determined using the promega CellID system.
See Supplementary File 4

Supplementary Table S3: Overview of genetic alterations in major CRC signaling pathways.
See Supplementary File 5

Supplementary Table S4: Cancer hotspot panel v2 targets.
See Supplementary File 6

Supplementary Table S5: Cell line characteristics and mutation profiles.
See Supplementary File 7
