# Supplementary material for: Characterization of novel low passage primary and metastatic colorectal cancer cell lines
Source: Oncotarget. 2016 Feb 15;7(12):14499–509. doi: 10.18632/oncotarget.7391 (PMC4924731; doi:10.18632/oncotarget.7391)
Supplement: Supplementary file 3 [file oncotarget-07-14499-s003.pdf]

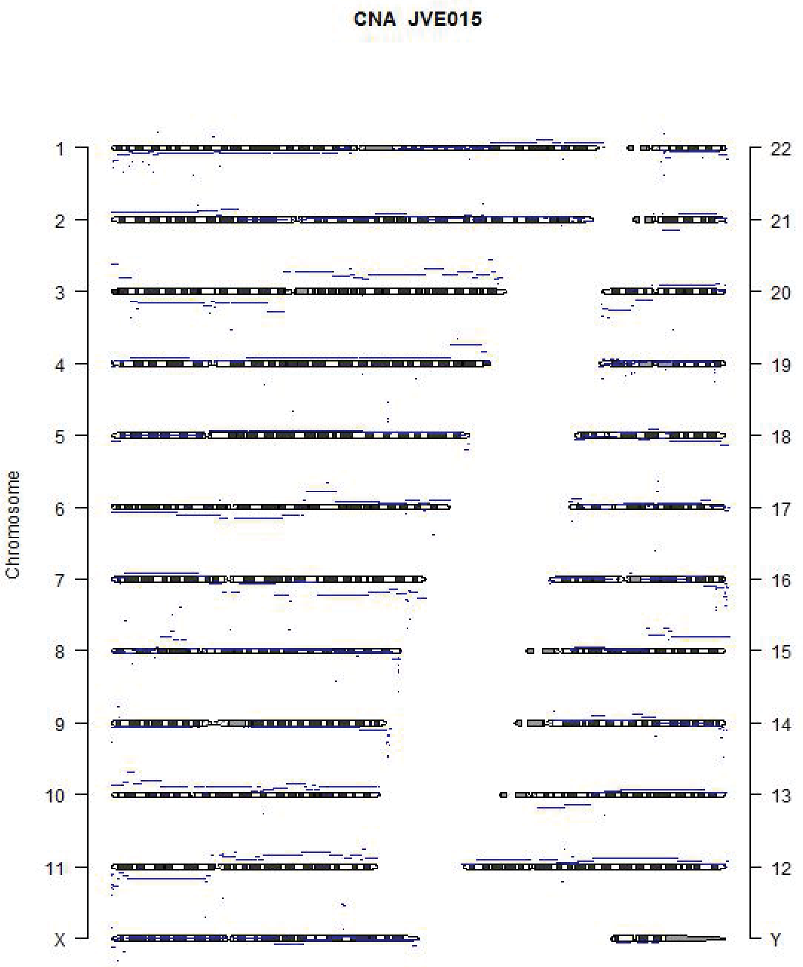
CNA JVE015
Chromosome
1
2
3
4
5
6
7
8
9
10
11
X
22
21
20
19
18
17
16
15
14
13
12
Y

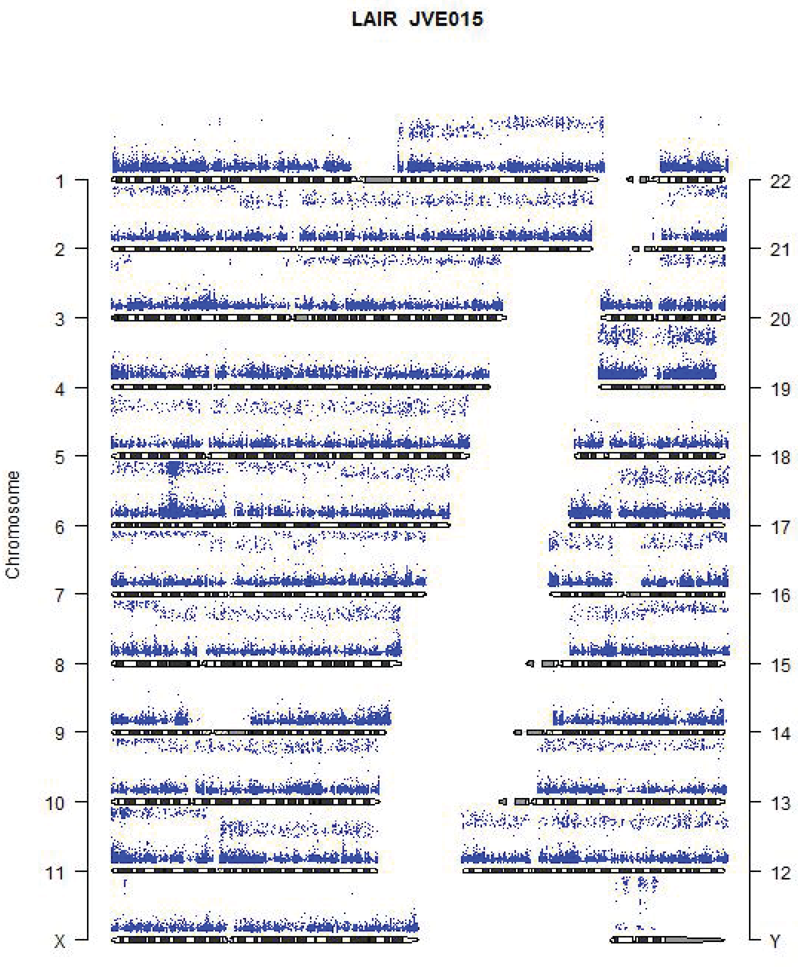
LAIR JVE015
Chromosome
1
2
3
4
5
6
7
8
9
10
11
X
22
21
20
19
18
17
16
15
14
13
12
Y

CNA JVE044

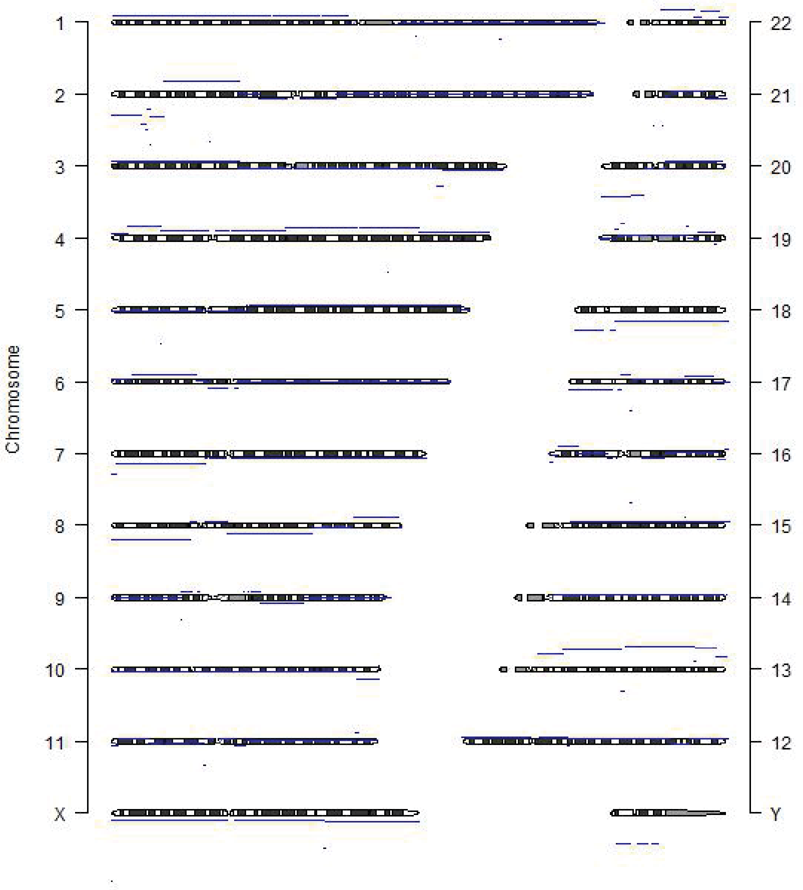

LAIR JVE044

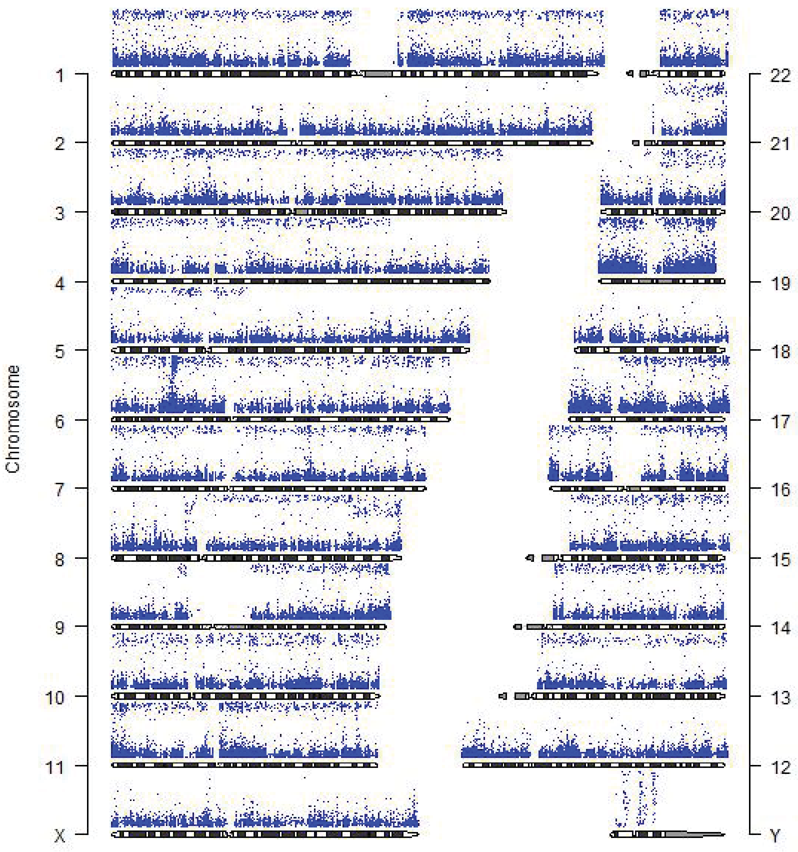

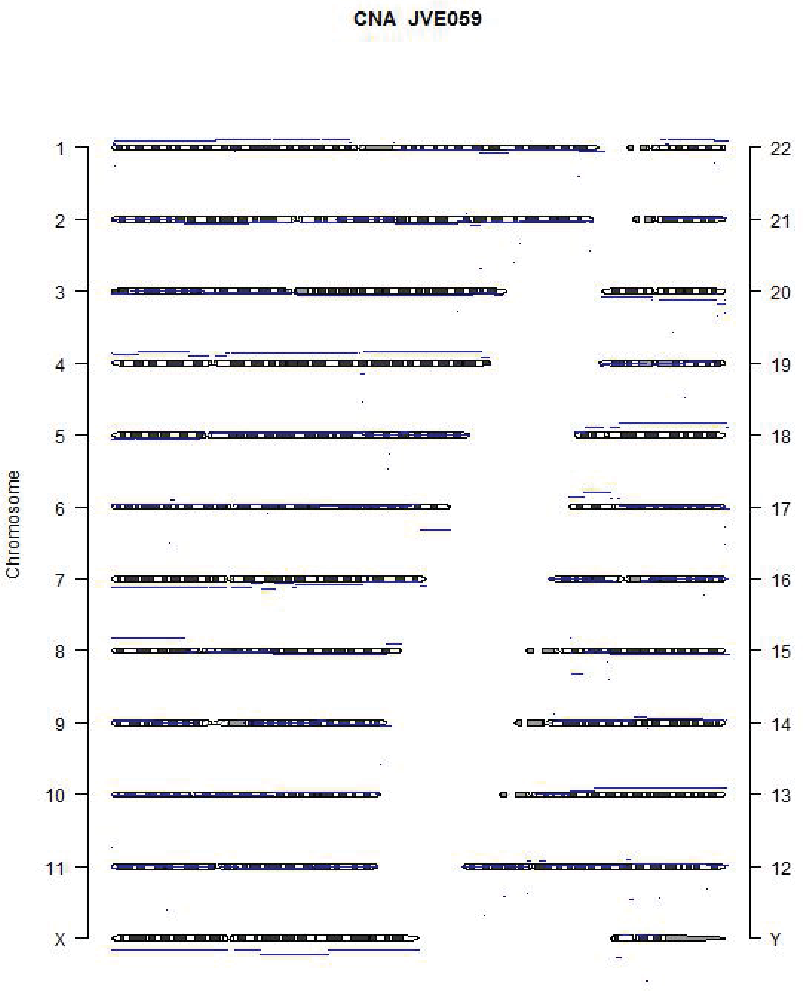

CNA JVE059
Chromosome
1
2
3
4
5
6
7
8
9
10
11
X
22
21
20
19
18
17
16
15
14
13
12
Y

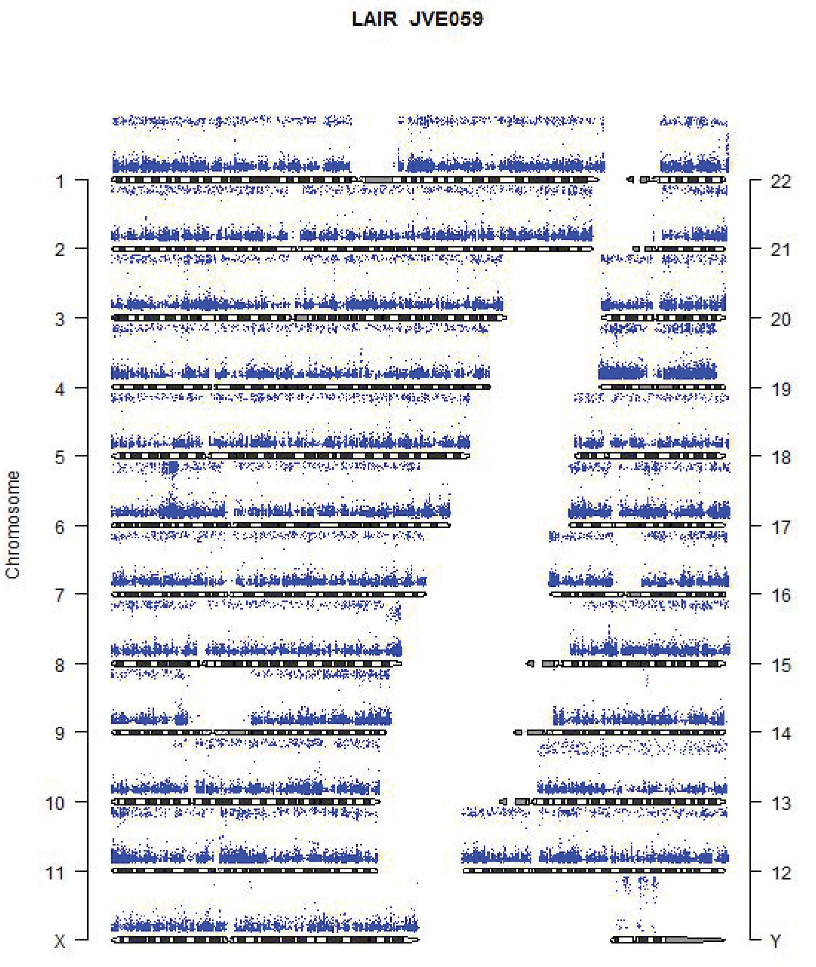

LAIR JVE059
Chromosome
1
2
3
4
5
6
7
8
9
10
11
X
22
21
20
19
18
17
16
15
14
13
12
Y

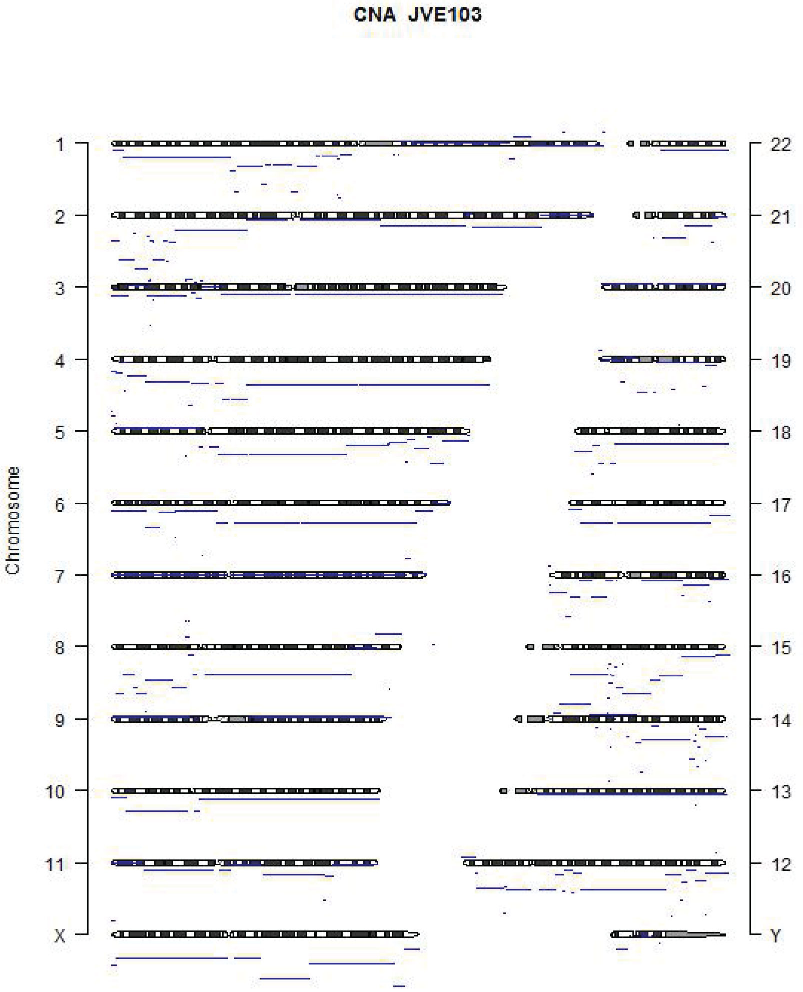
CNA JVE103
Chromosome
1
2
3
4
5
6
7
8
9
10
11
X
22
21
20
19
18
17
16
15
14
13
12
Y

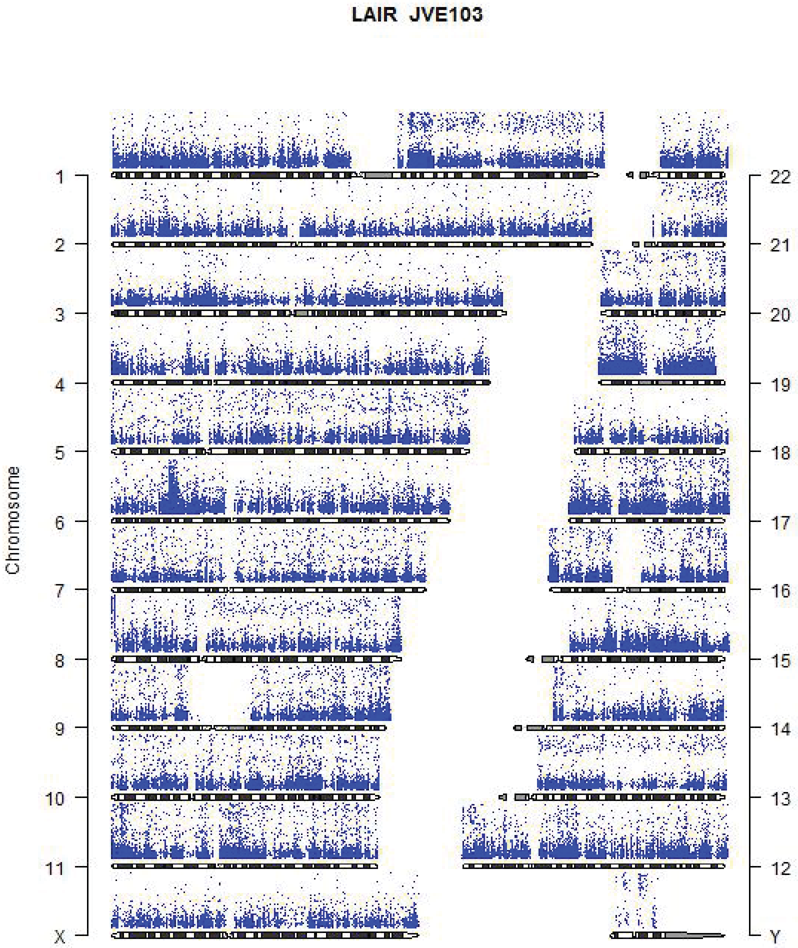
LAIR JVE103
Chromosome
1
2
3
4
5
6
7
8
9
10
11
X
22
21
20
19
18
17
16
15
14
13
12
Y

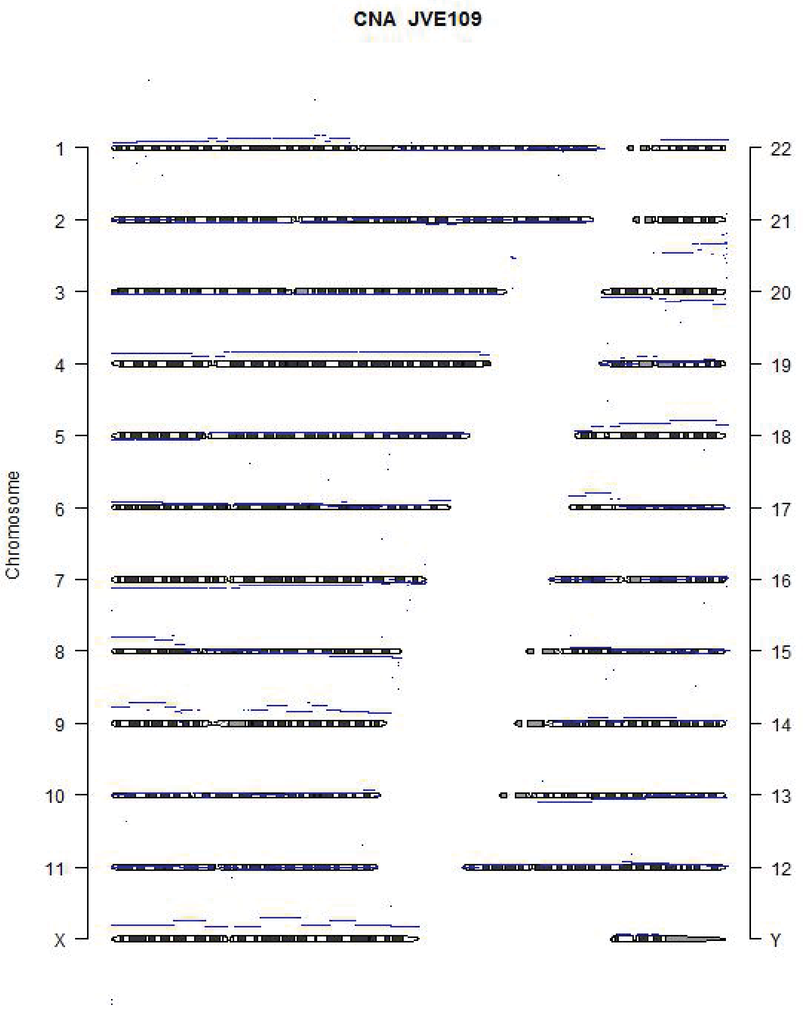
CNA JVE109
Chromosome
1
2
3
4
5
6
7
8
9
10
11
X
22
21
20
19
18
17
16
15
14
13
12
Y

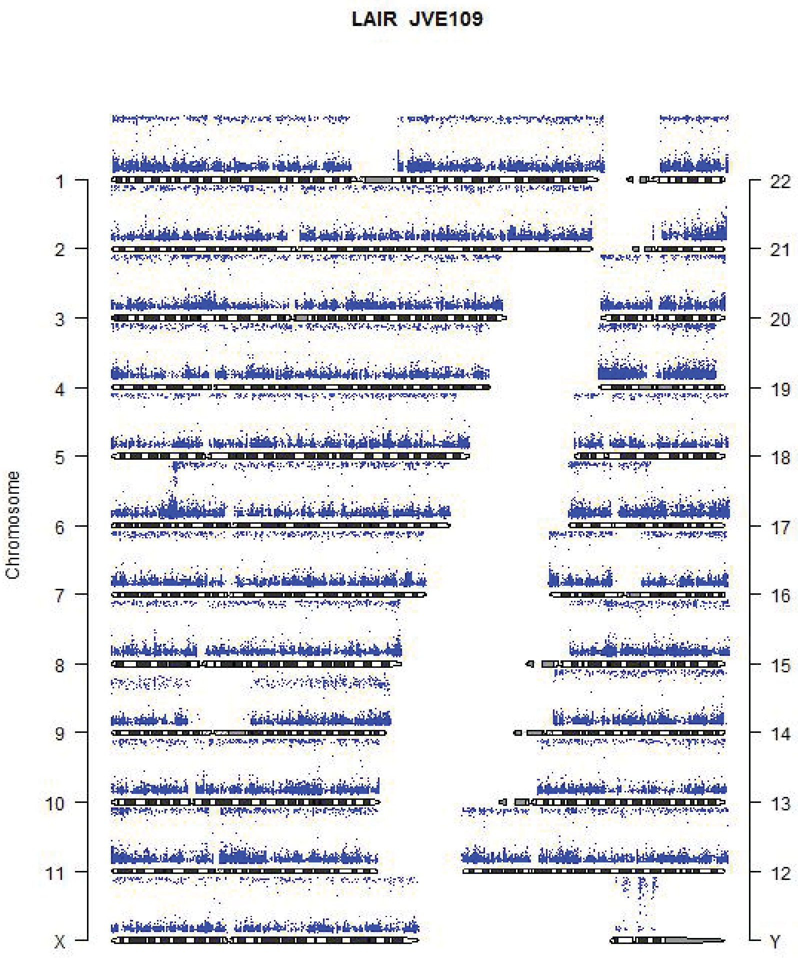
LAIR JVE109
Chromosome
1
2
3
4
5
6
7
8
9
10
11
X
22
21
20
19
18
17
16
15
14
13
12
Y

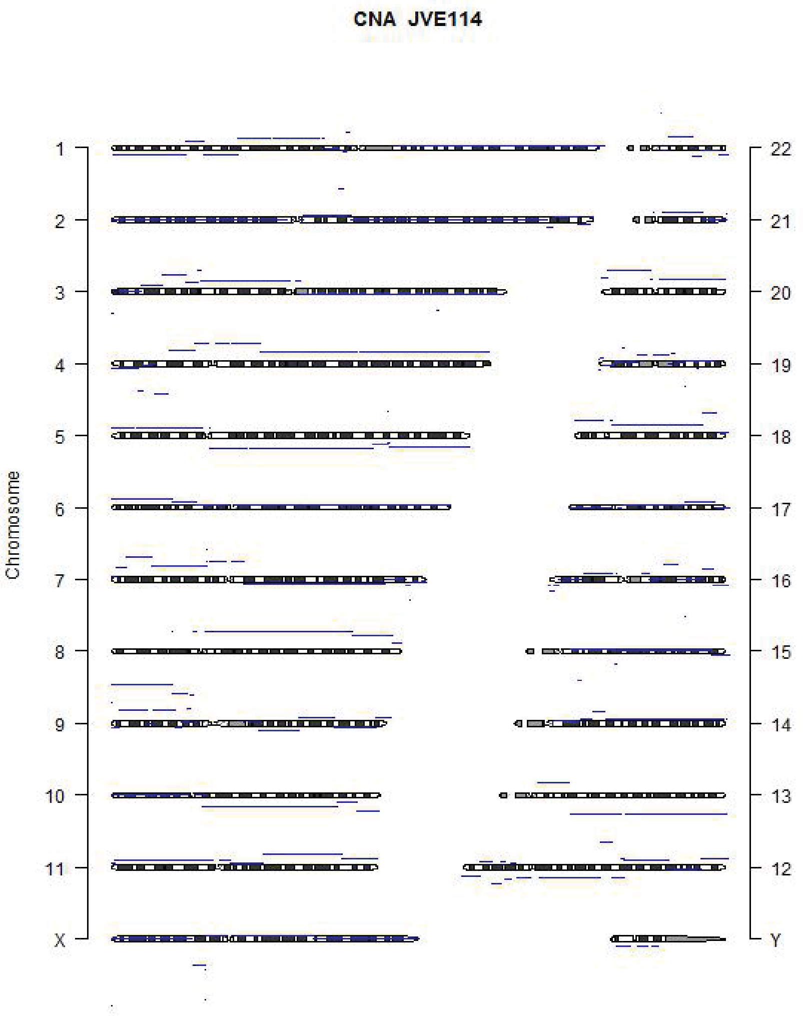
CNA JVE114
Chromosome
1
2
3
4
5
6
7
8
9
10
11
X
22
21
20
19
18
17
16
15
14
13
12
Y

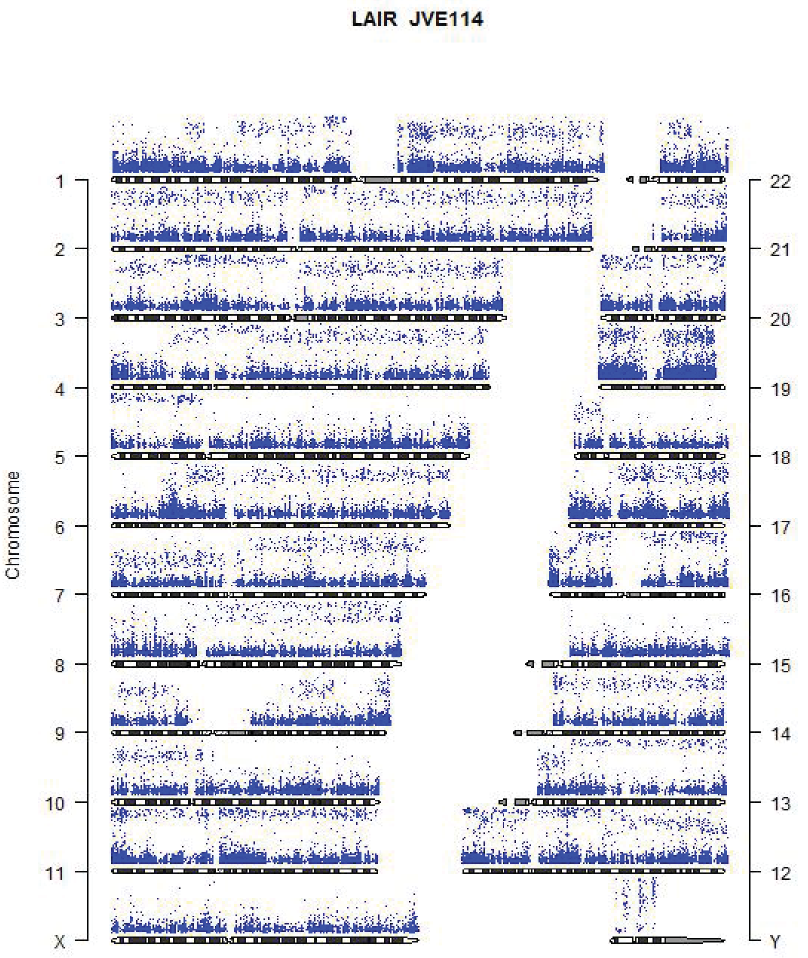
LAIR JVE114
Chromosome
1
2
3
4
5
6
7
8
9
10
11
X
22
21
20
19
18
17
16
15
14
13
12
Y

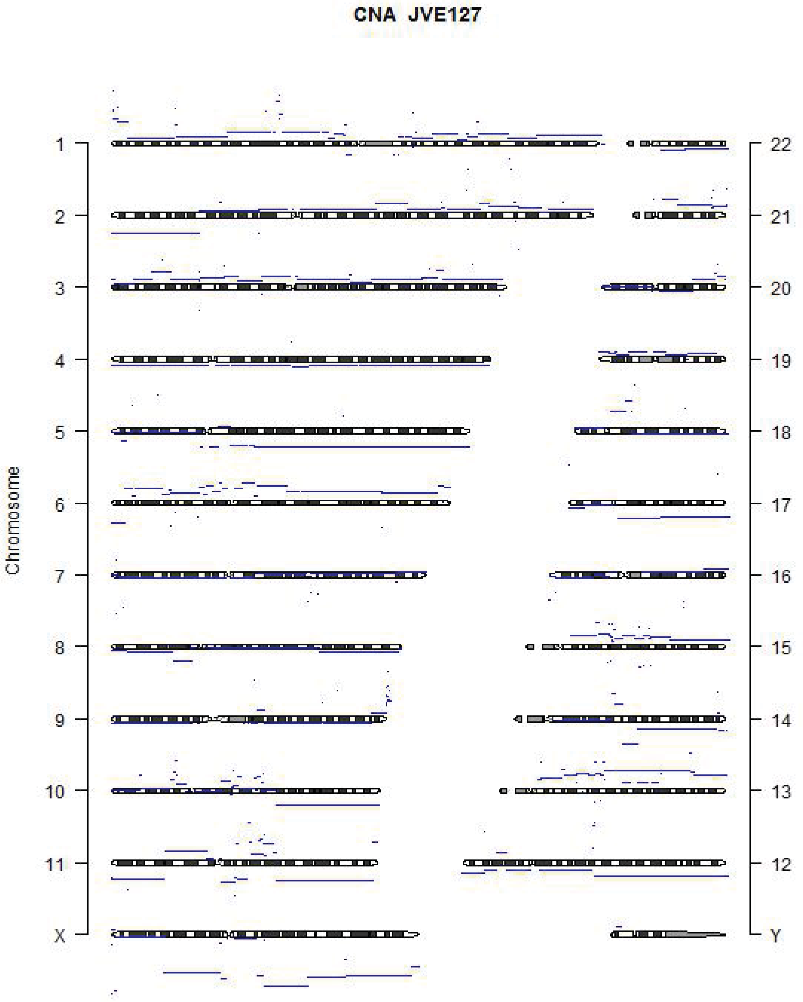
CNA JVE127
Chromosome
1
2
3
4
5
6
7
8
9
10
11
X
22
21
20
19
18
17
16
15
14
13
12
Y

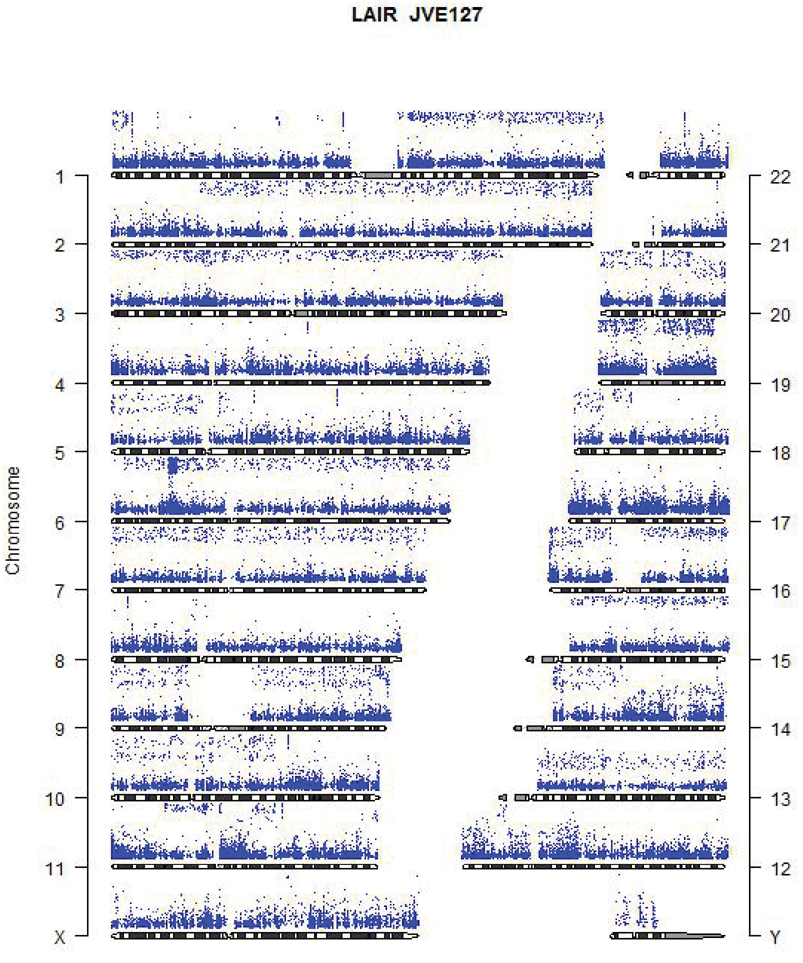
LAIR JVE127
Chromosome
1
2
3
4
5
6
7
8
9
10
11
X
22
21
20
19
18
17
16
15
14
13
12
Y

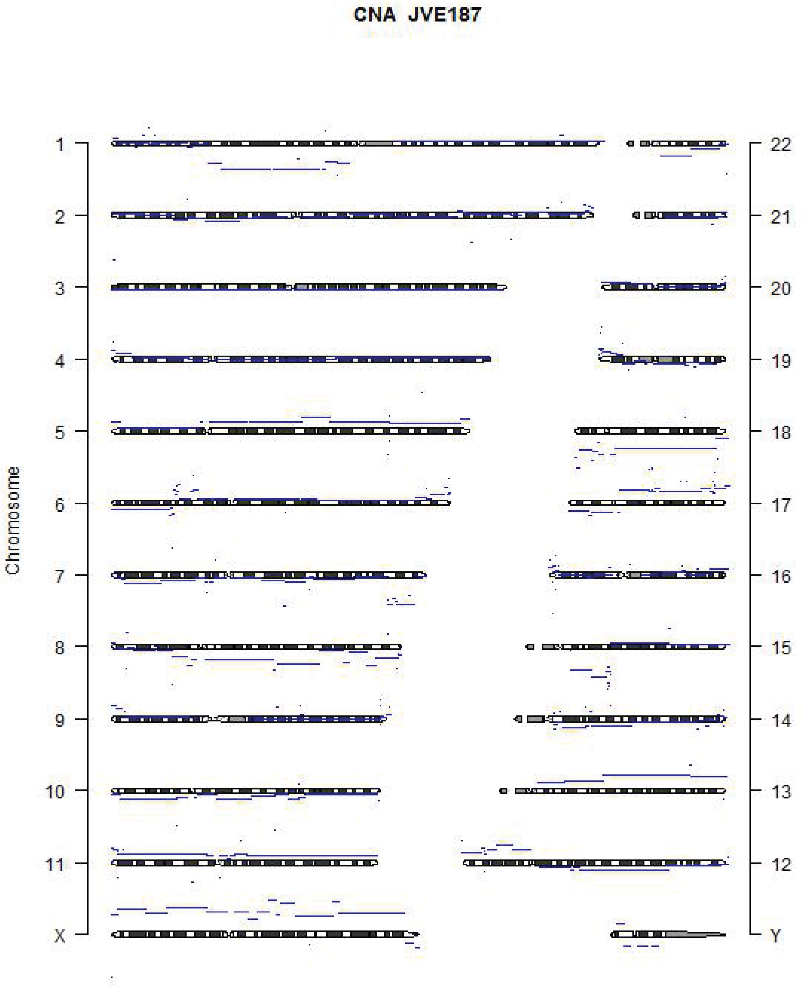
CNA JVE187
Chromosome
1
2
3
4
5
6
7
8
9
10
11
X
22
21
20
19
18
17
16
15
14
13
12
Y

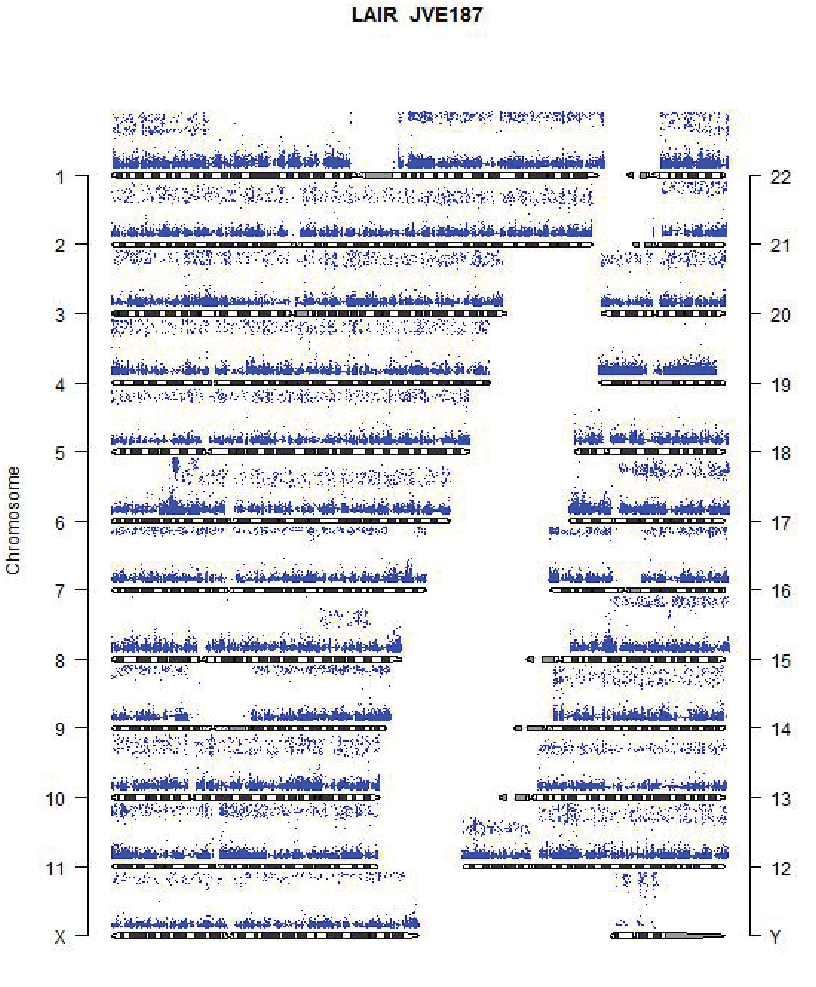
LAIR JVE187
Chromosome
1
2
3
4
5
6
7
8
9
10
11
X
22
21
20
19
18
17
16
15
14
13
12
Y

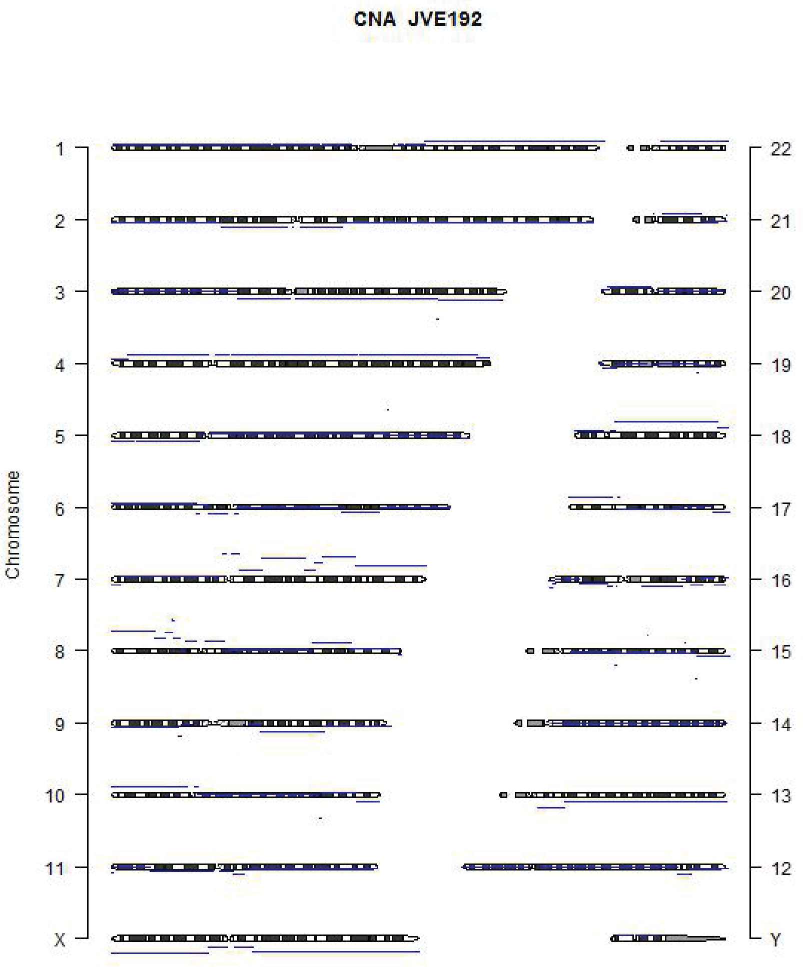
CNA JVE192
Chromosome
1
2
3
4
5
6
7
8
9
10
11
X
22
21
20
19
18
17
16
15
14
13
12
Y

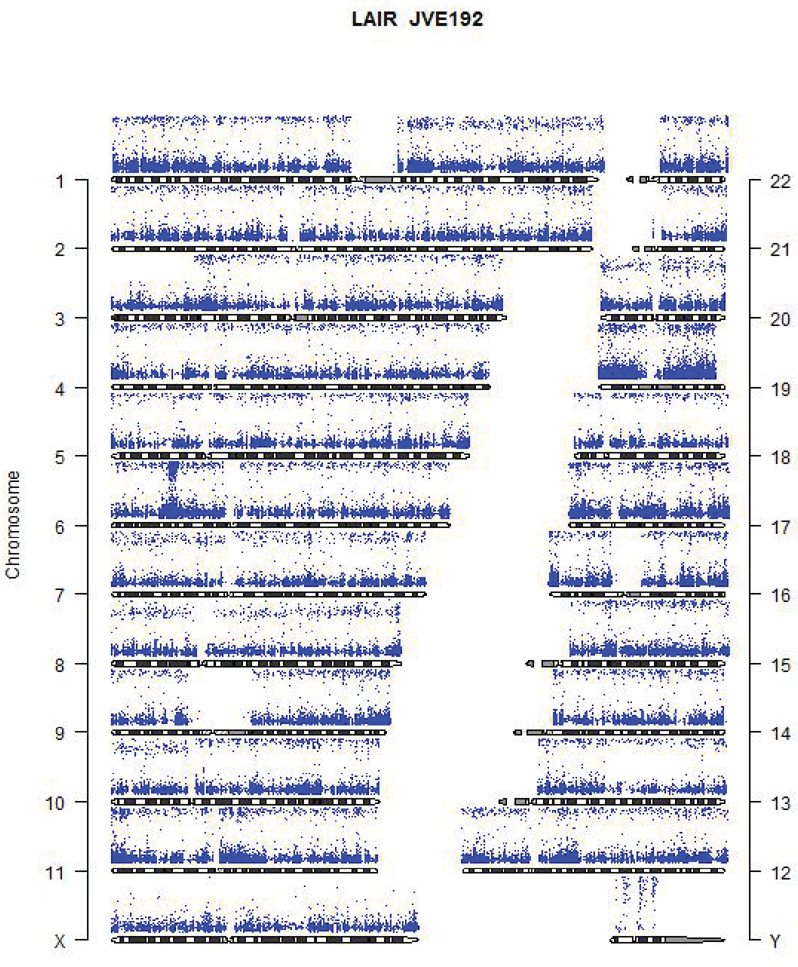
LAIR JVE192
Chromosome
1
2
3
4
5
6
7
8
9
10
11
X
22
21
20
19
18
17
16
15
14
13
12
Y

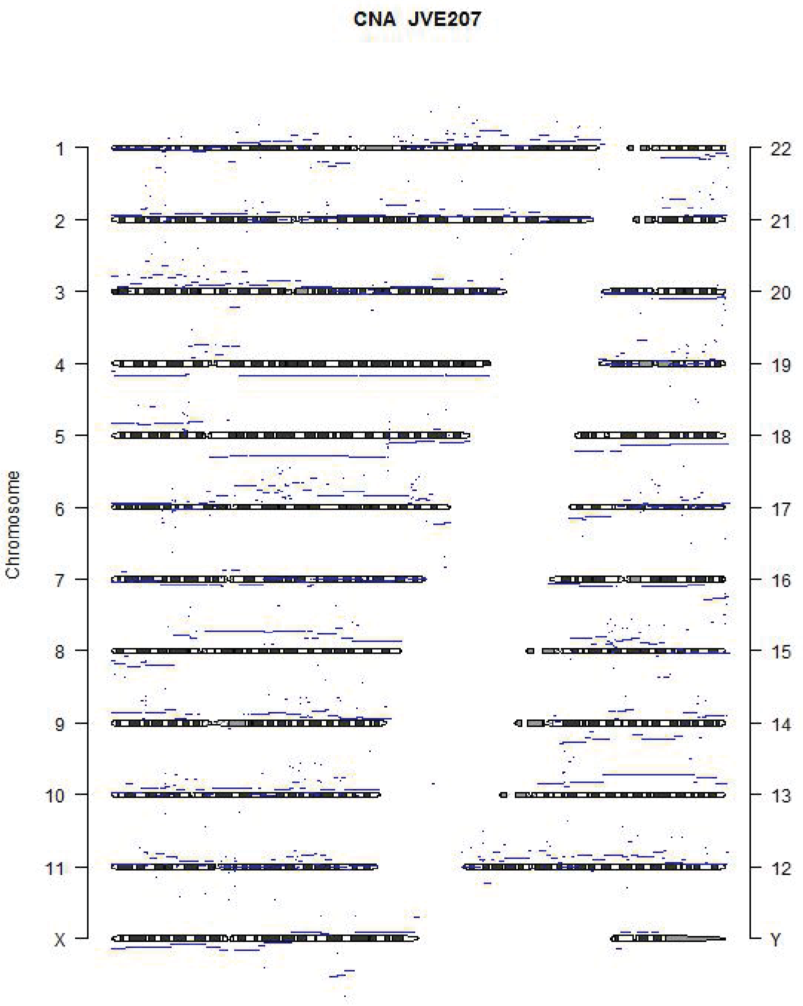
CNA JVE207
Chromosome
1
2
3
4
5
6
7
8
9
10
11
X
22
21
20
19
18
17
16
15
14
13
12
Y

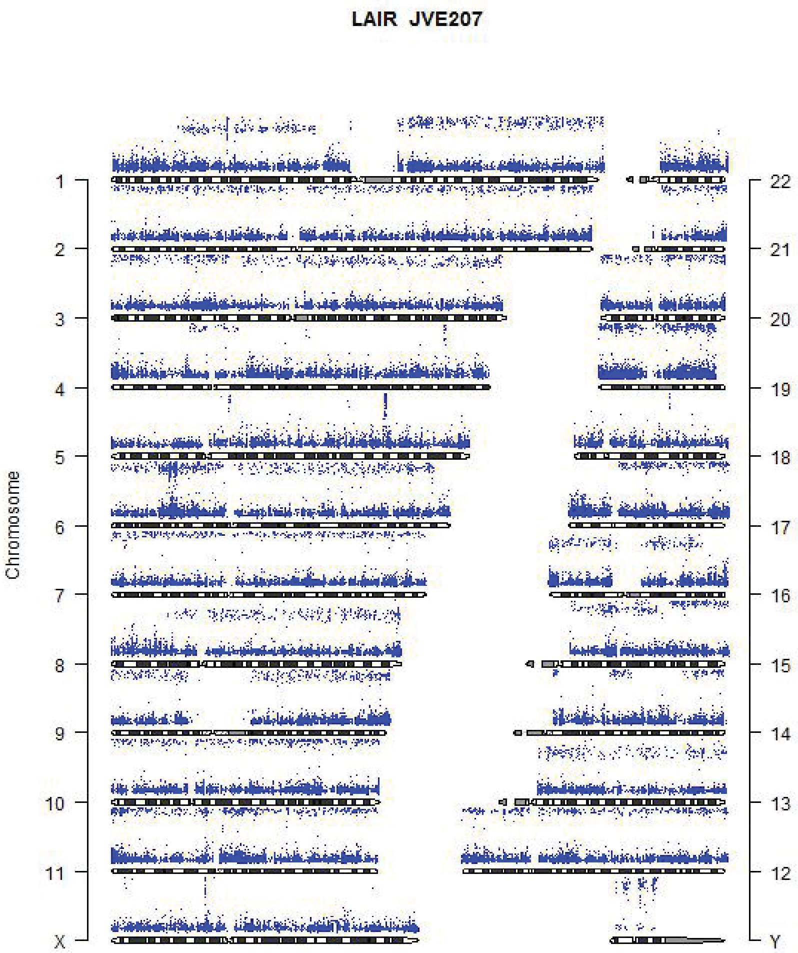
LAIR JVE207
Chromosome
1
2
3
4
5
6
7
8
9
10
11
X
22
21
20
19
18
17
16
15
14
13
12
Y

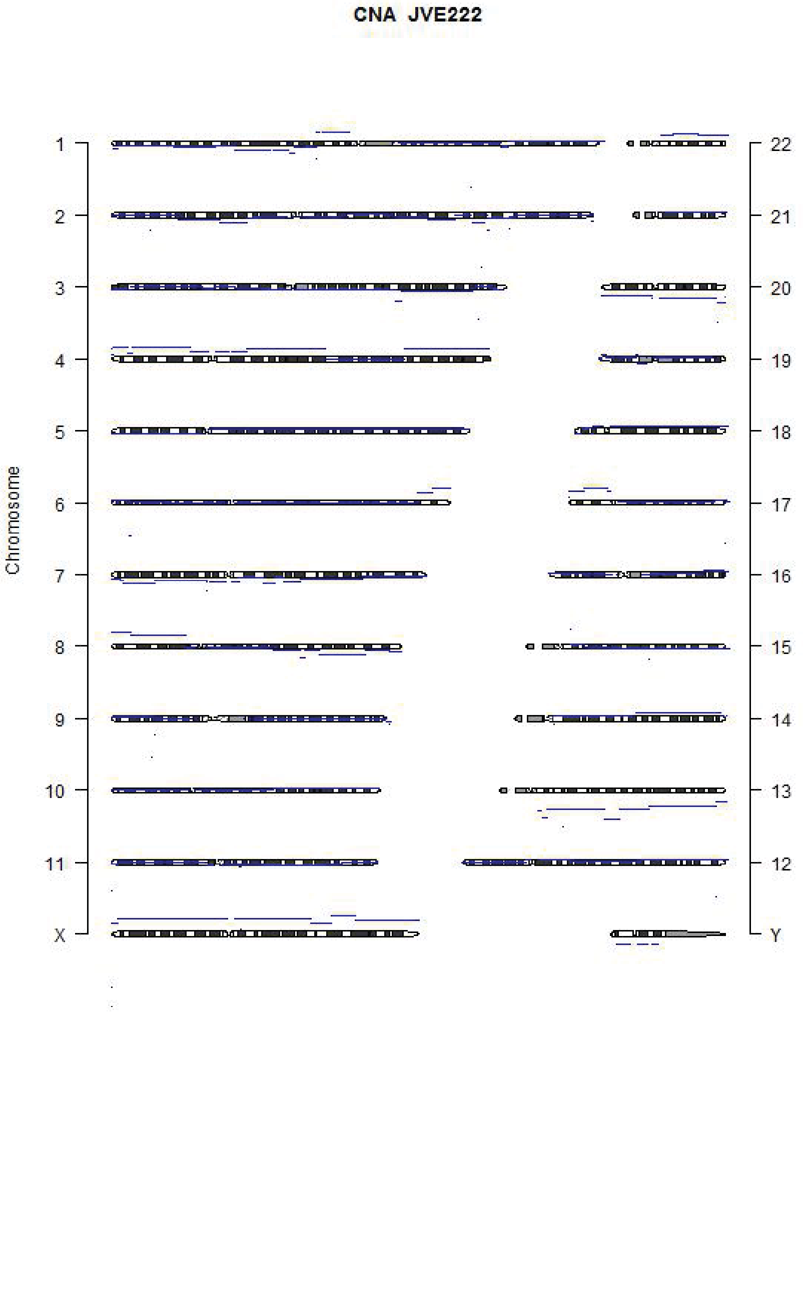
CNA JVE222
Chromosome
1
2
3
4
5
6
7
8
9
10
11
X
22
21
20
19
18
17
16
15
14
13
12
Y

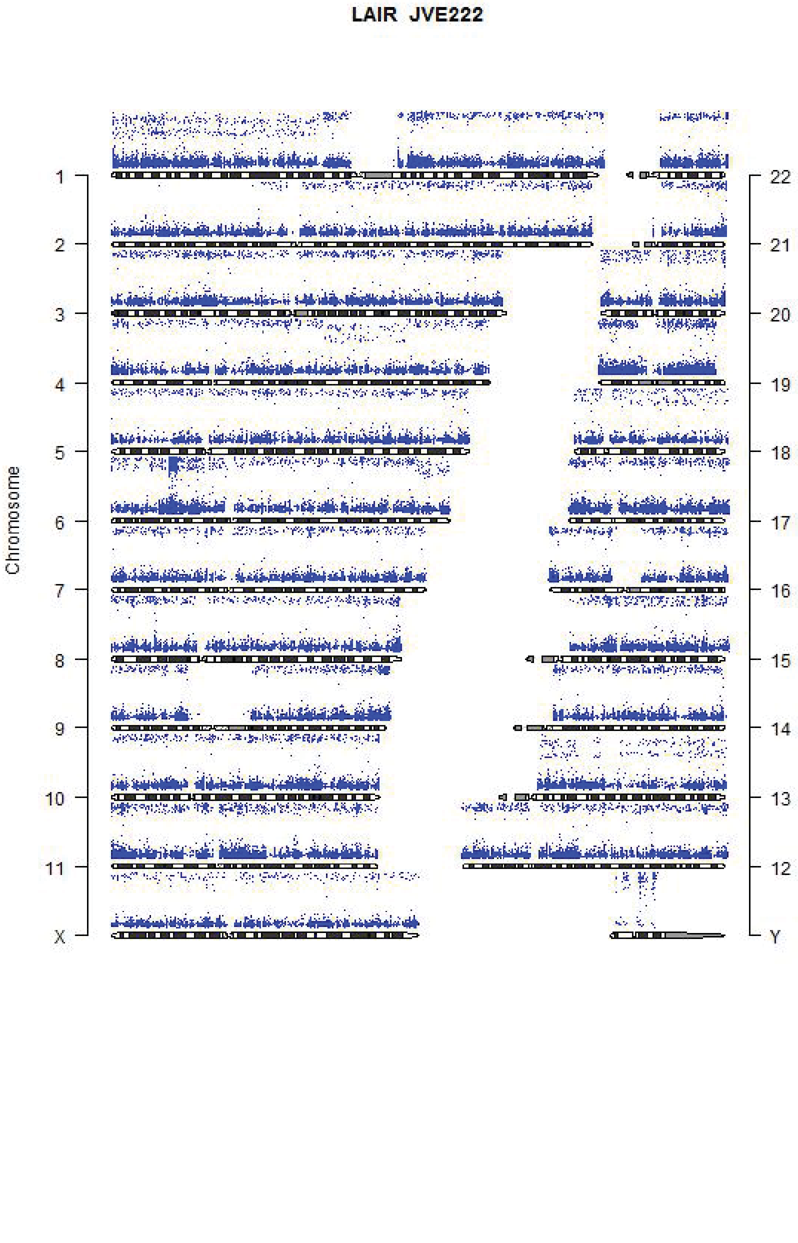
LAIR JVE222
Chromosome
1
2
3
4
5
6
7
8
9
10
11
X
22
21
20
19
18
17
16
15
14
13
12
Y

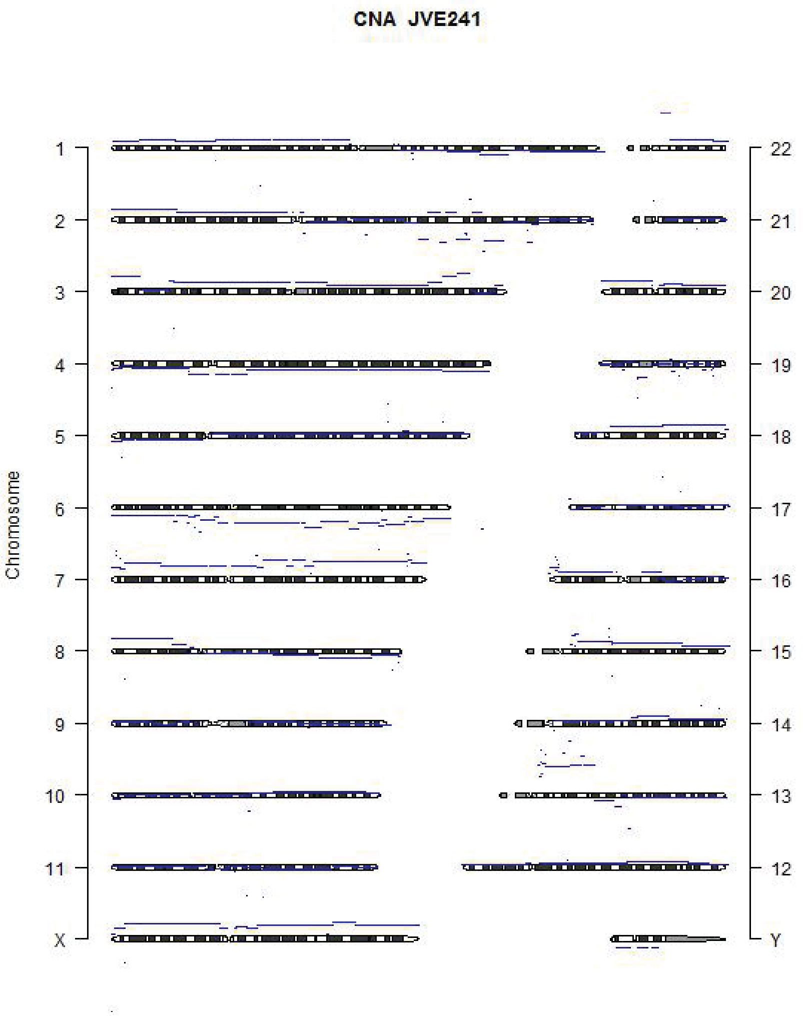
CNA JVE241
Chromosome
1
2
3
4
5
6
7
8
9
10
11
X
22
21
20
19
18
17
16
15
14
13
12
Y

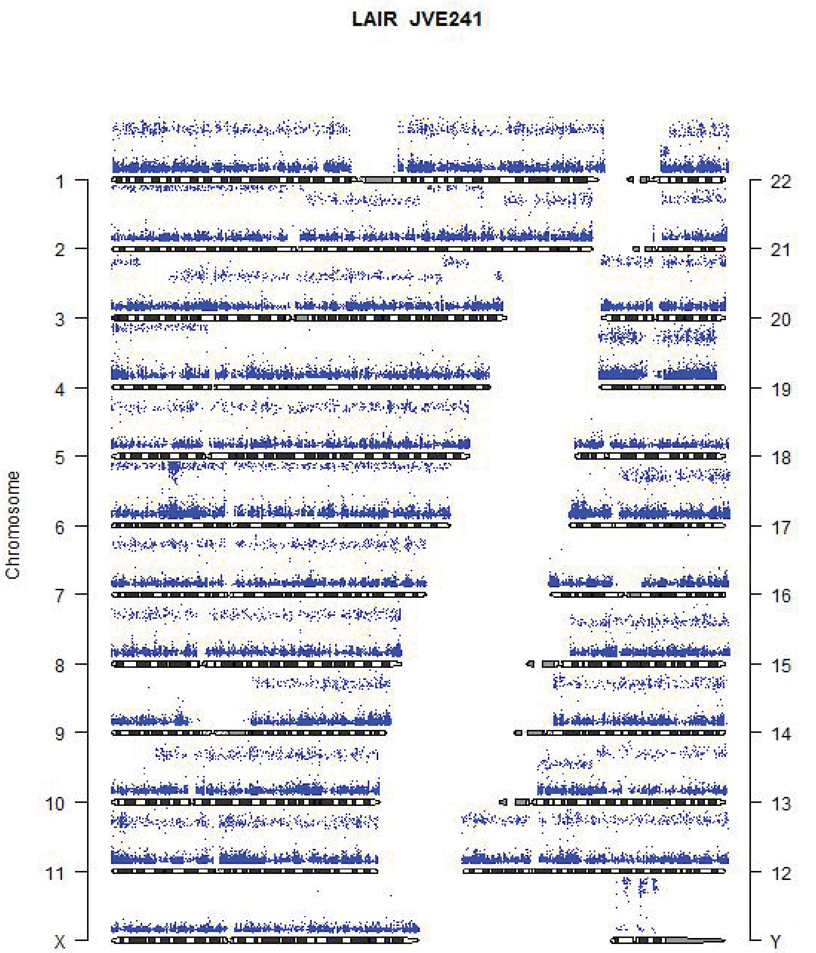
LAIR JVE241
Chromosome
1
2
3
4
5
6
7
8
9
10
11
X
22
21
20
19
18
17
16
15
14
13
12
Y

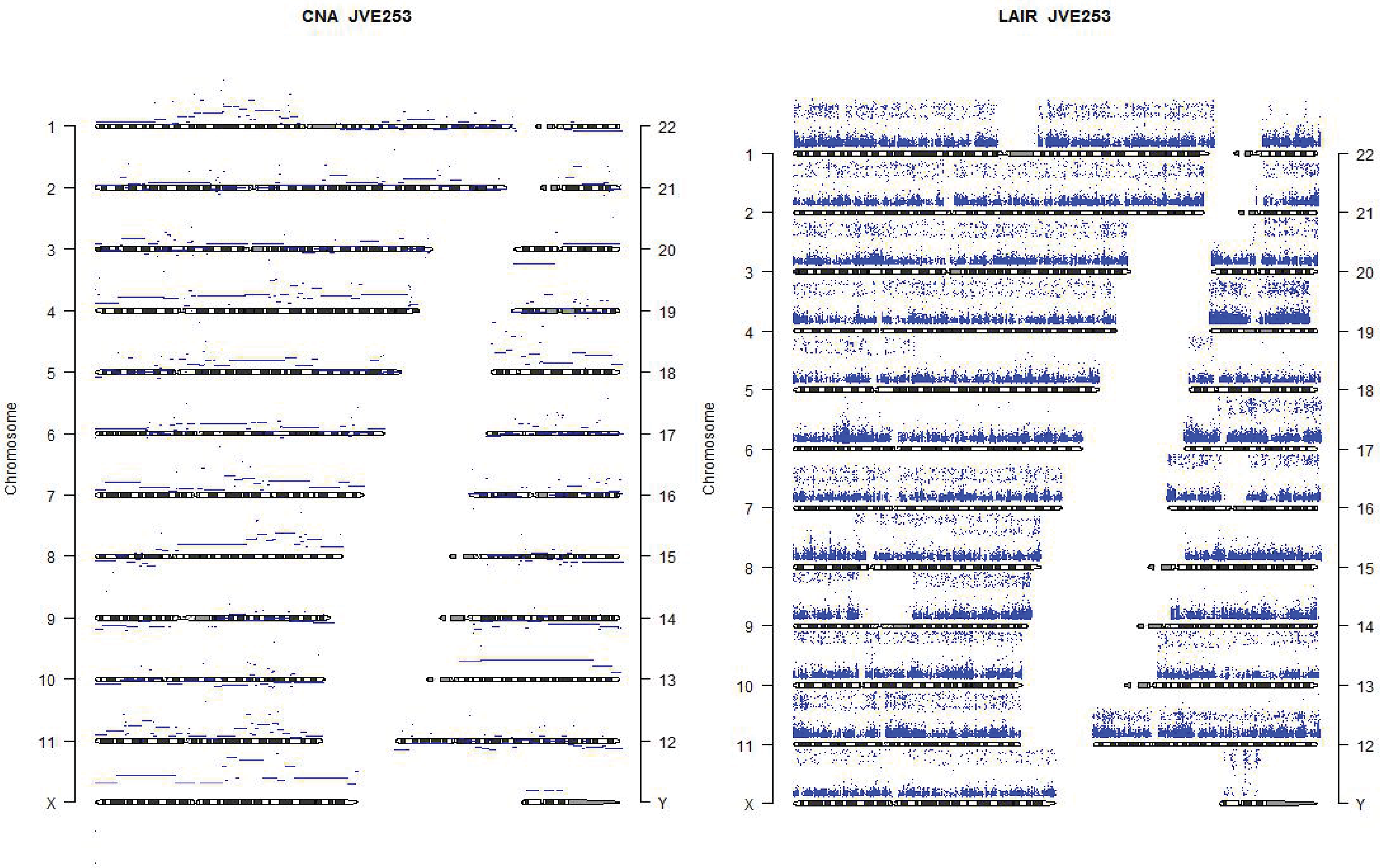
CNA JVE253
LAIR JVE253
Chromosome
1
2
3
4
5
6
7
8
9
10
11
X
22
21
20
19
18
17
16
15
14
13
12
Y

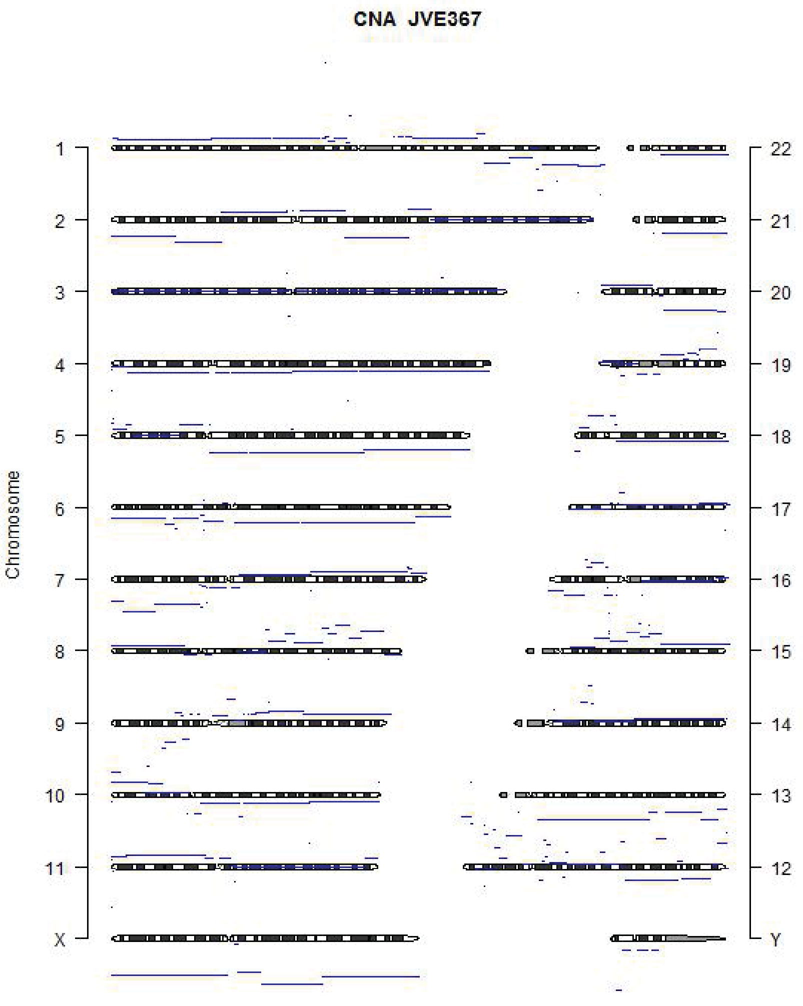
CNA JVE367
Chromosome
1
2
3
4
5
6
7
8
9
10
11
X
22
21
20
19
18
17
16
15
14
13
12
Y

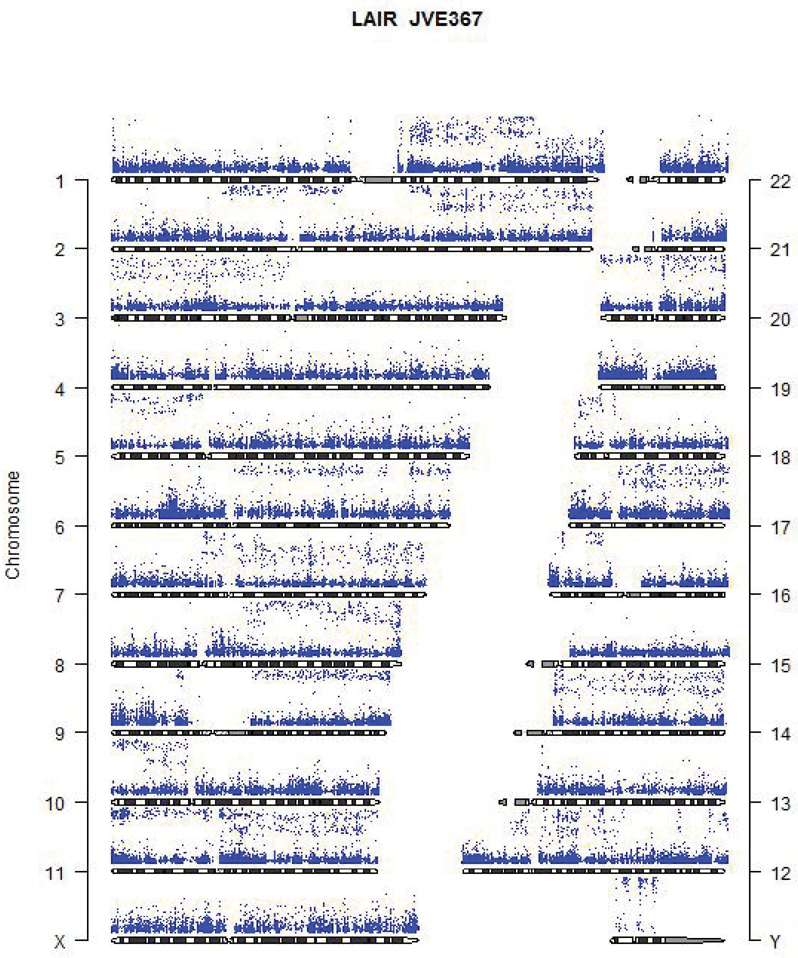
LAIR JVE367
Chromosome
1
2
3
4
5
6
7
8
9
10
11
X
22
21
20
19
18
17
16
15
14
13
12
Y

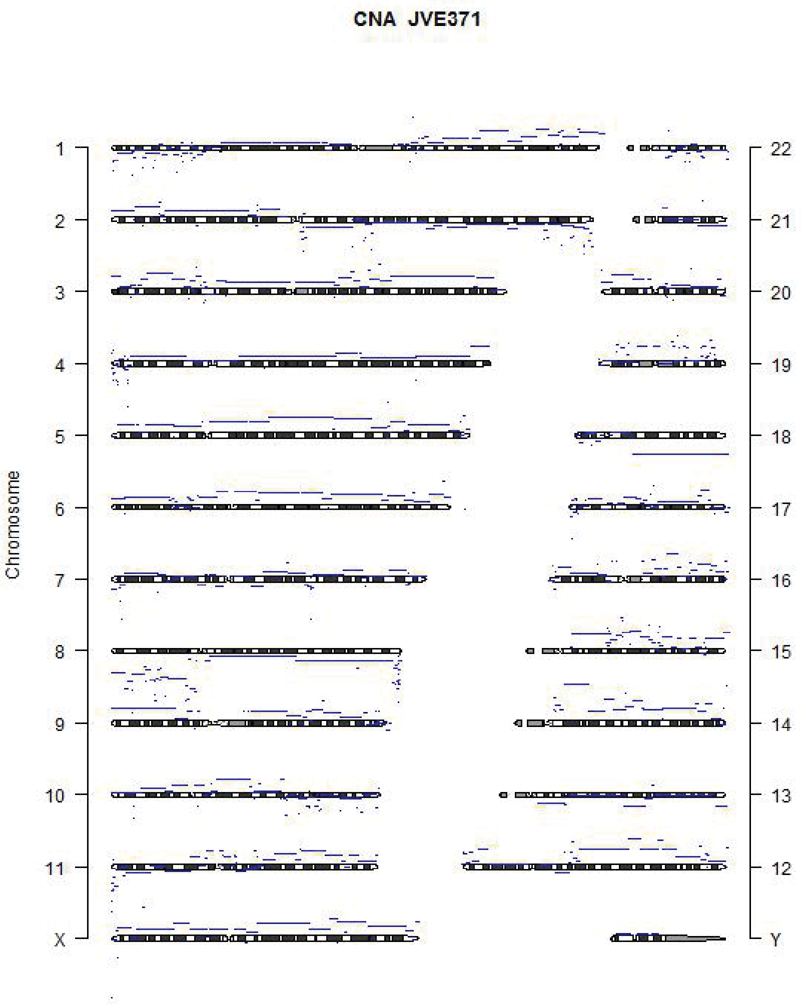
CNA JVE371
Chromosome
1
2
3
4
5
6
7
8
9
10
11
X
22
21
20
19
18
17
16
15
14
13
12
Y

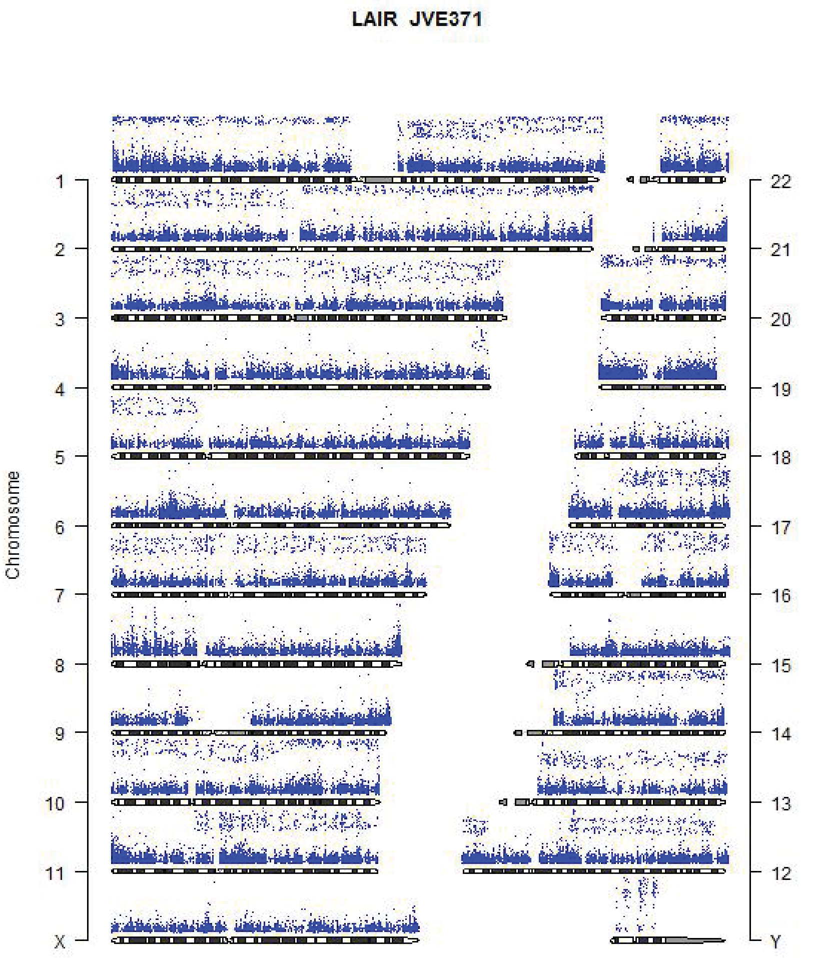
LAIR JVE371
Chromosome
1
2
3
4
5
6
7
8
9
10
11
X
22
21
20
19
18
17
16
15
14
13
12
Y

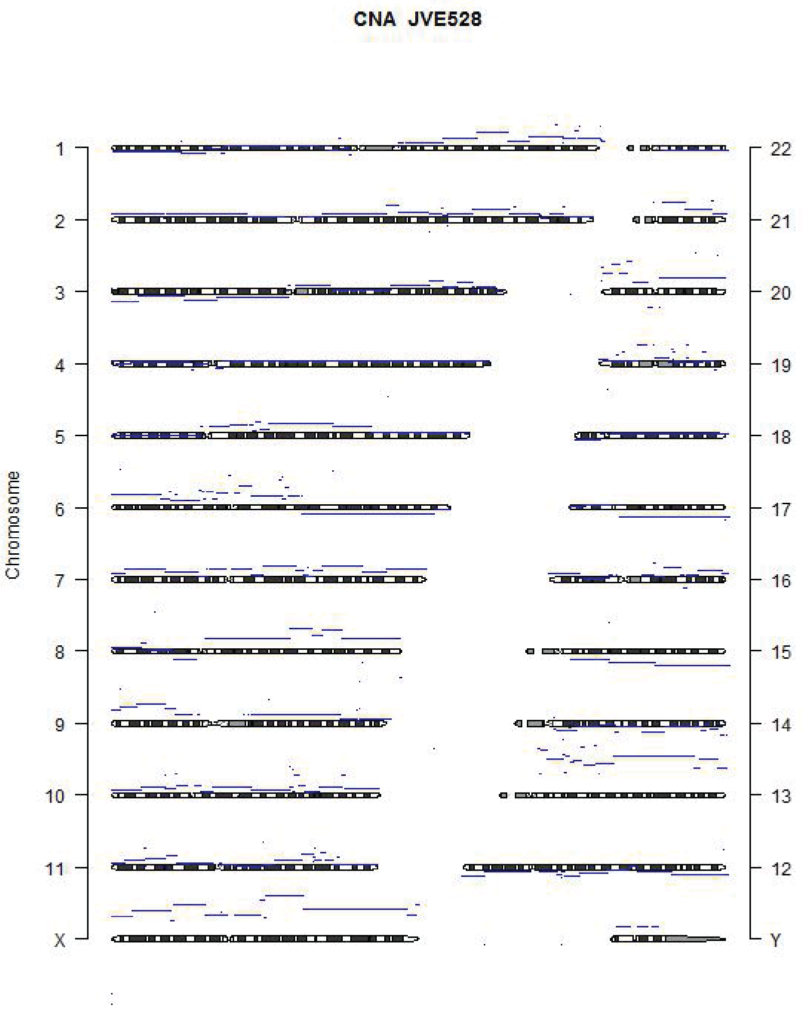
CNA JVE528
Chromosome
1
2
3
4
5
6
7
8
9
10
11
X
22
21
20
19
18
17
16
15
14
13
12
Y

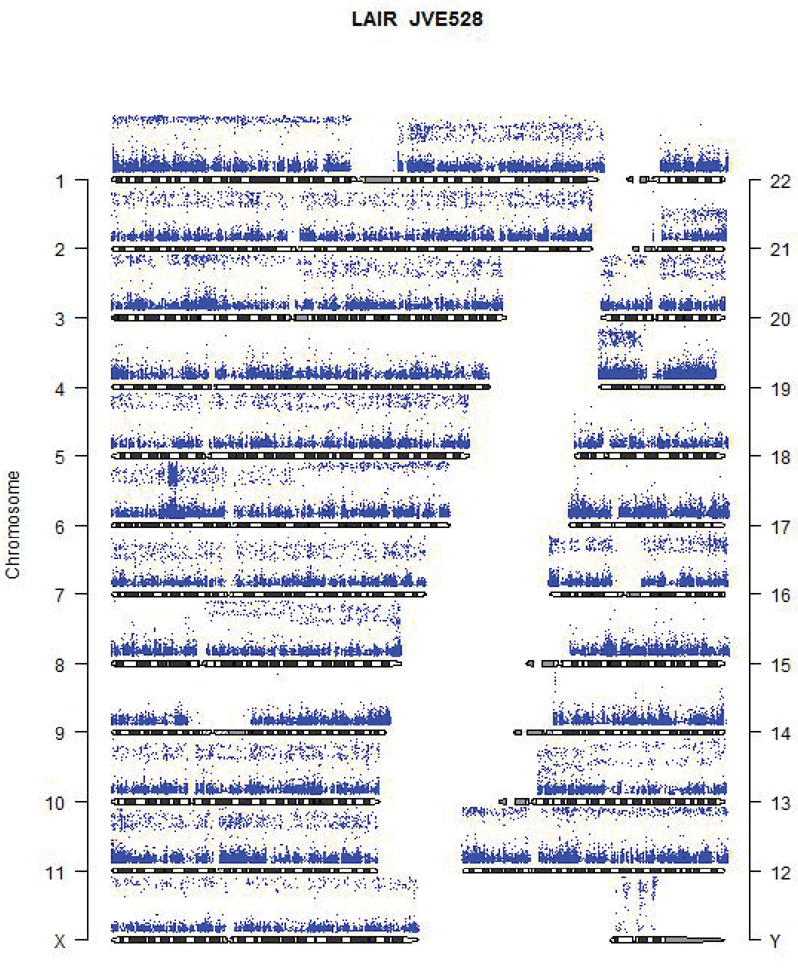
LAIR JVE528
Chromosome
1
2
3
4
5
6
7
8
9
10
11
X
22
21
20
19
18
17
16
15
14
13
12
Y

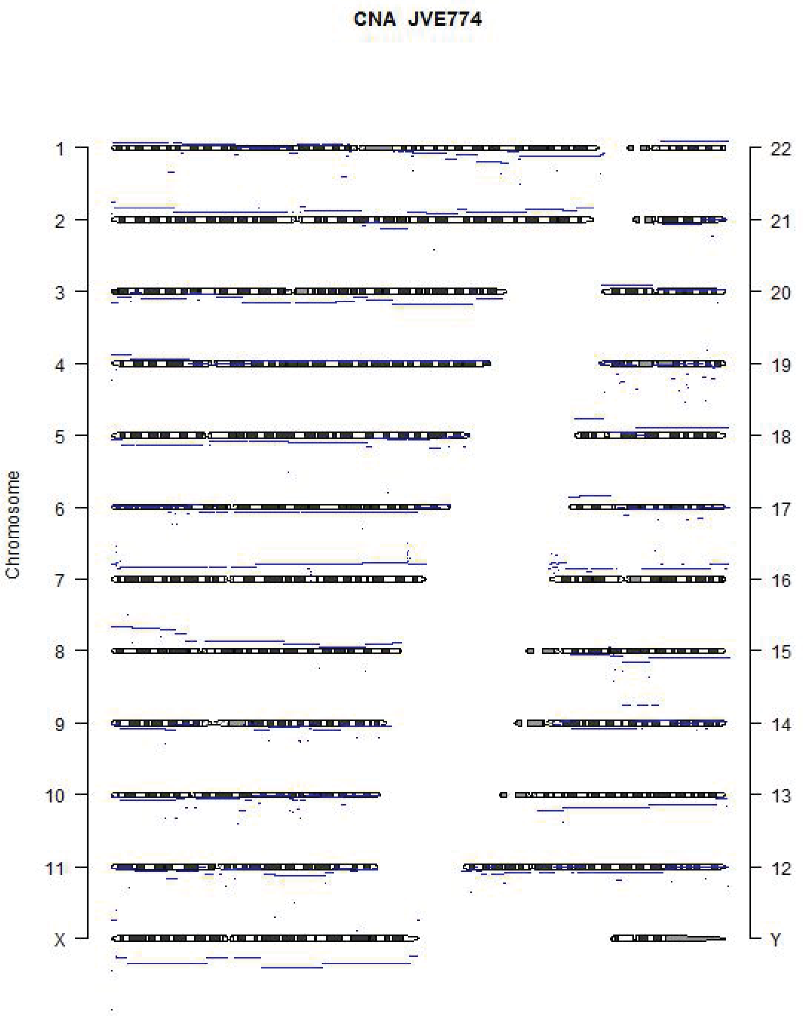
CNA JVE774
Chromosome
1
2
3
4
5
6
7
8
9
10
11
X
22
21
20
19
18
17
16
15
14
13
12
Y

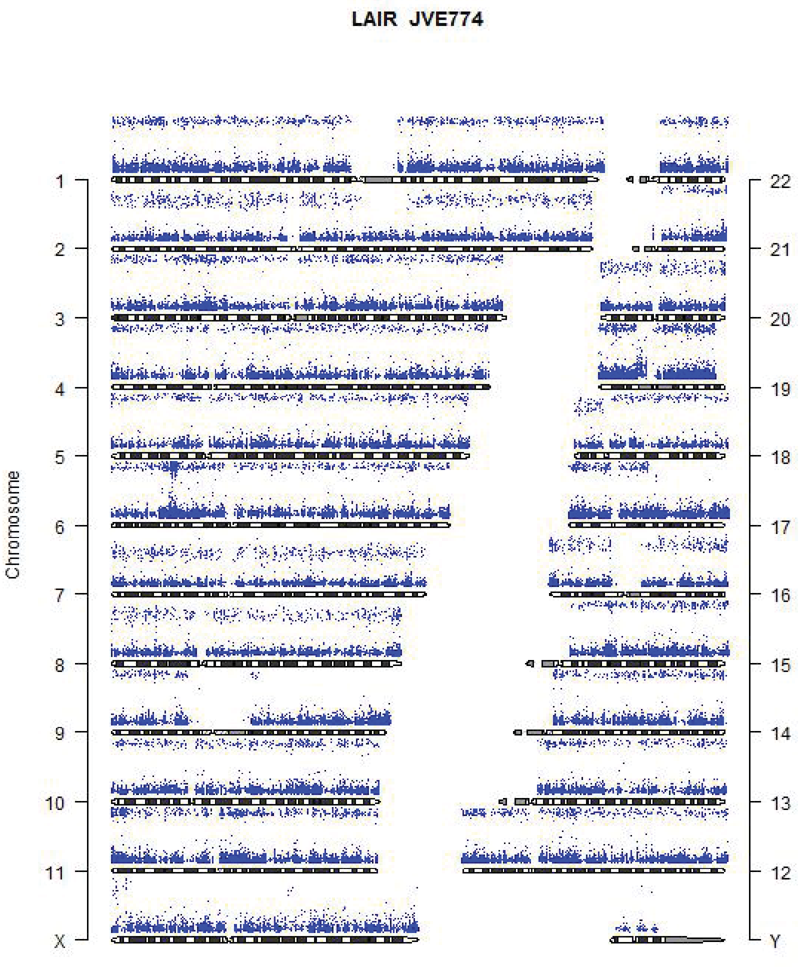
LAIR JVE774
Chromosome
1
2
3
4
5
6
7
8
9
10
11
X
22
21
20
19
18
17
16
15
14
13
12
Y

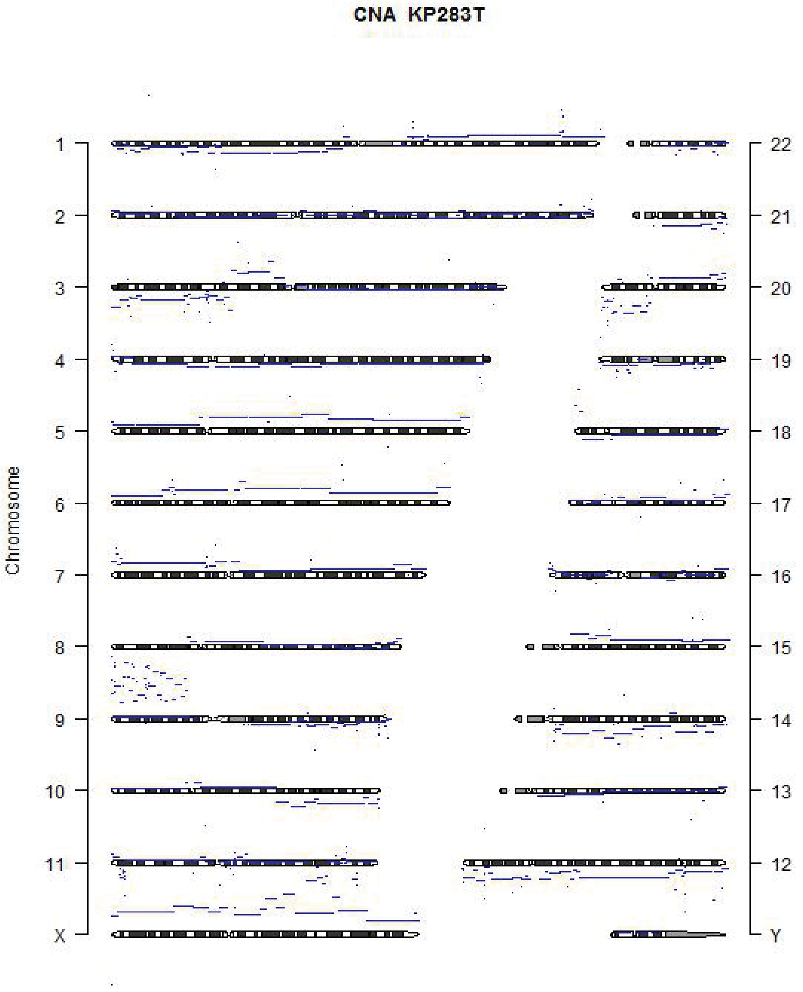
CNA KP283T
Chromosome
1
2
3
4
5
6
7
8
9
10
11
X
22
21
20
19
18
17
16
15
14
13
12
Y

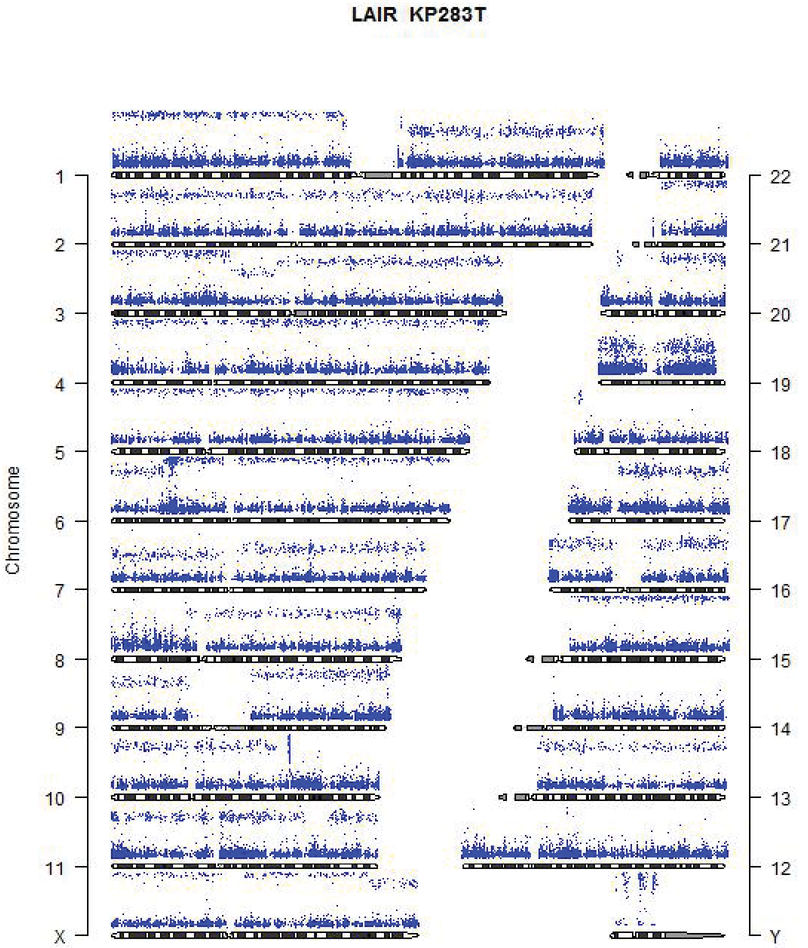
LAIR KP283T
Chromosome
1
2
3
4
5
6
7
8
9
10
11
X
22
21
20
19
18
17
16
15
14
13
12
Y

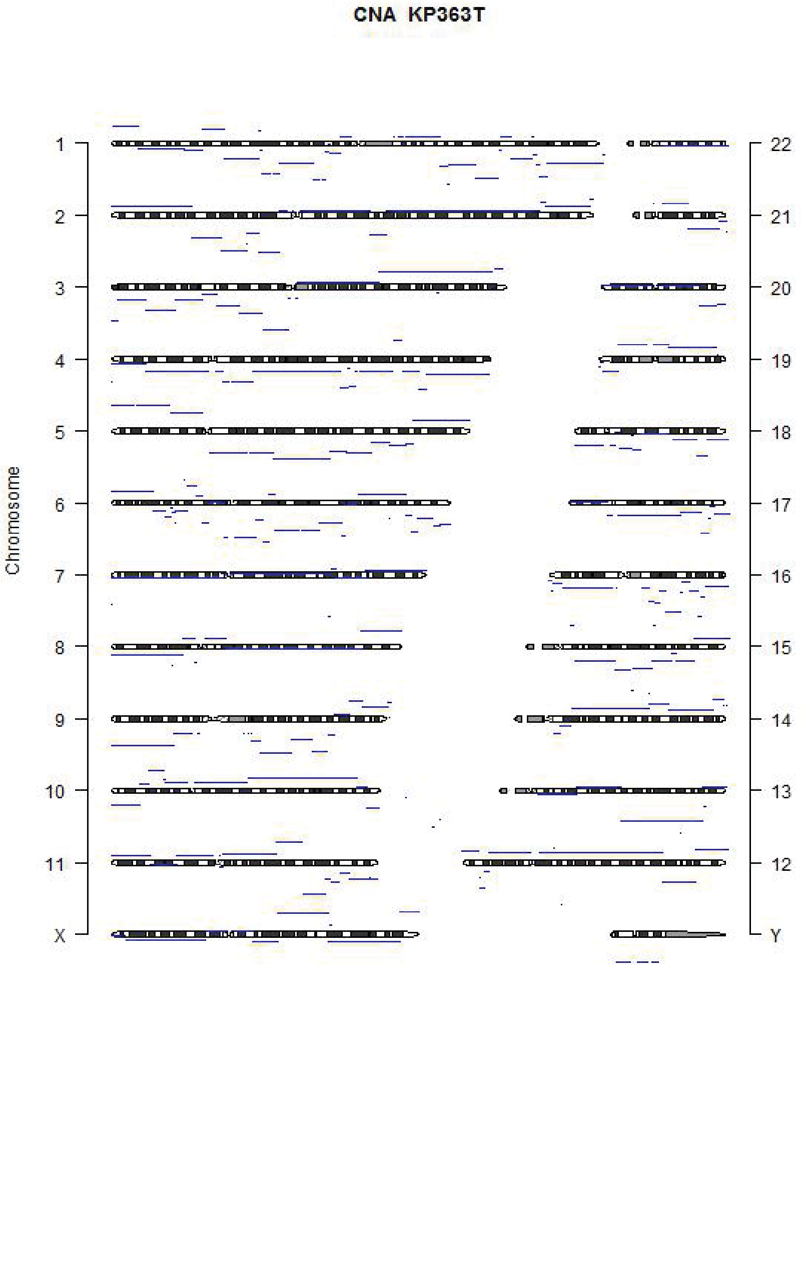
CNA KP363T
Chromosome
1
2
3
4
5
6
7
8
9
10
11
X
22
21
20
19
18
17
16
15
14
13
12
Y

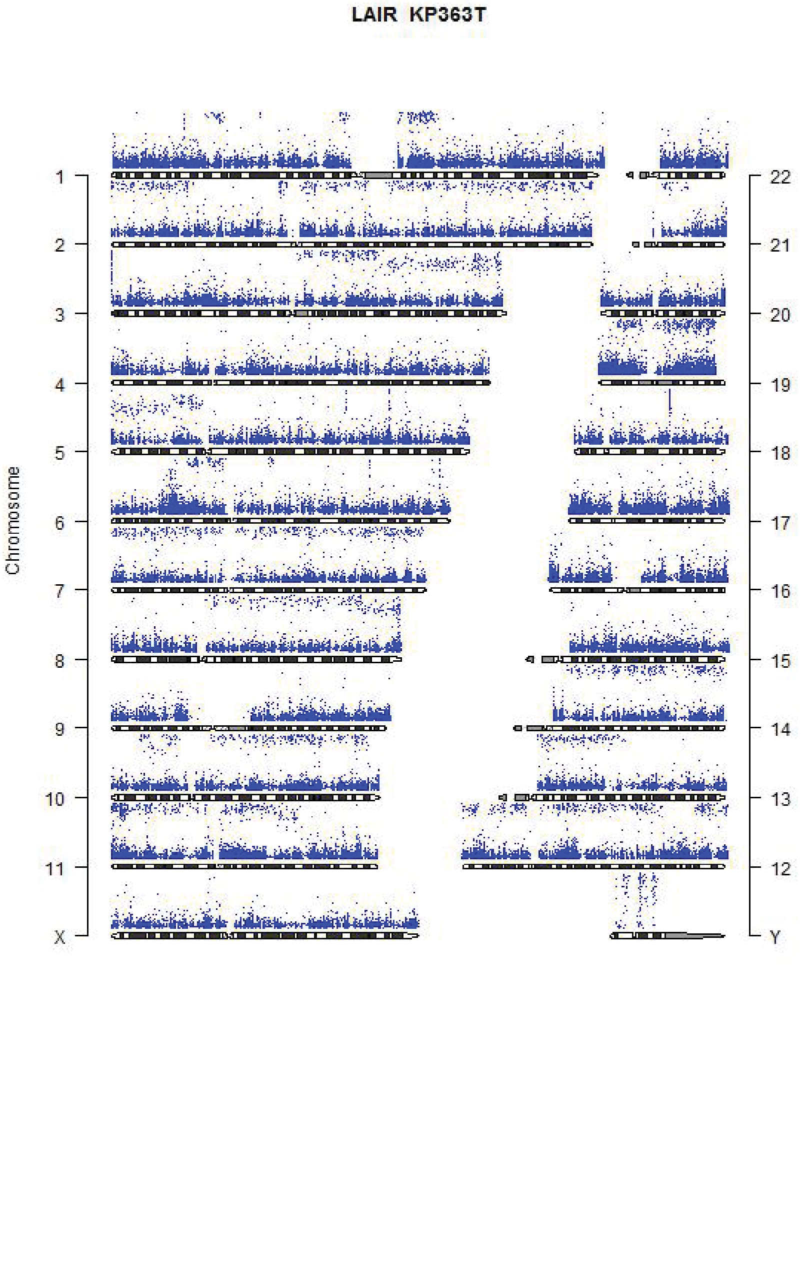
LAIR KP363T
Chromosome
1
2
3
4
5
6
7
8
9
10
11
X
22
21
20
19
18
17
16
15
14
13
12
Y

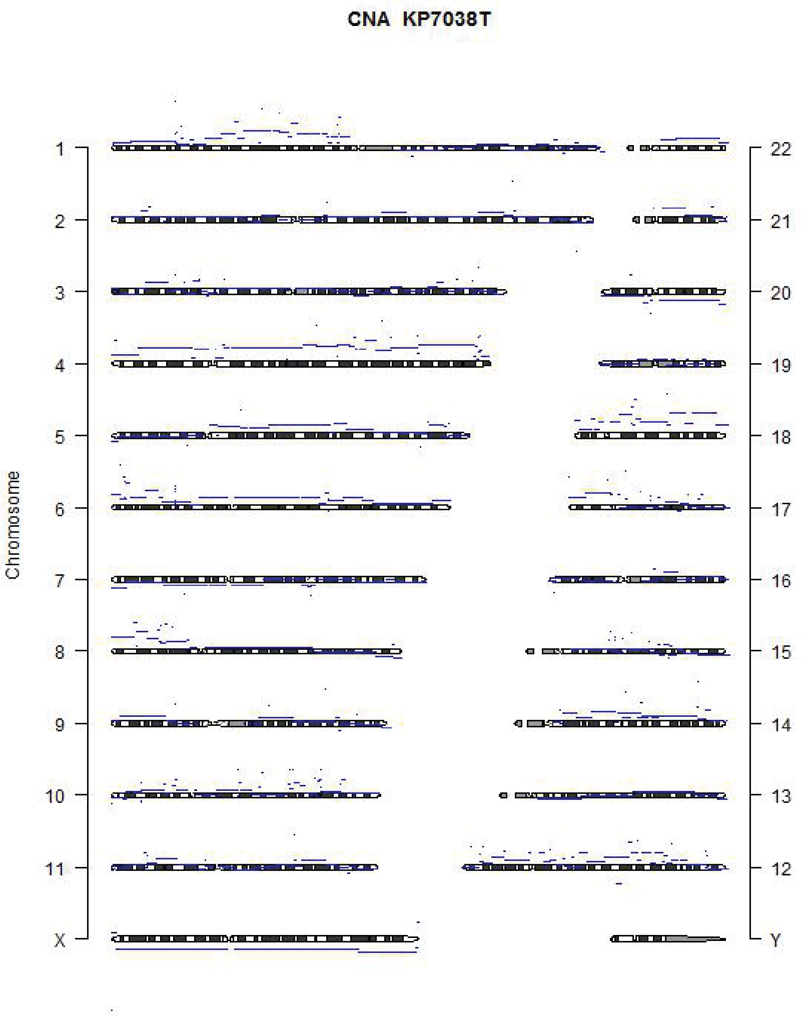
CNA KP7038T
Chromosome
1
2
3
4
5
6
7
8
9
10
11
X
22
21
20
19
18
17
16
15
14
13
12
Y

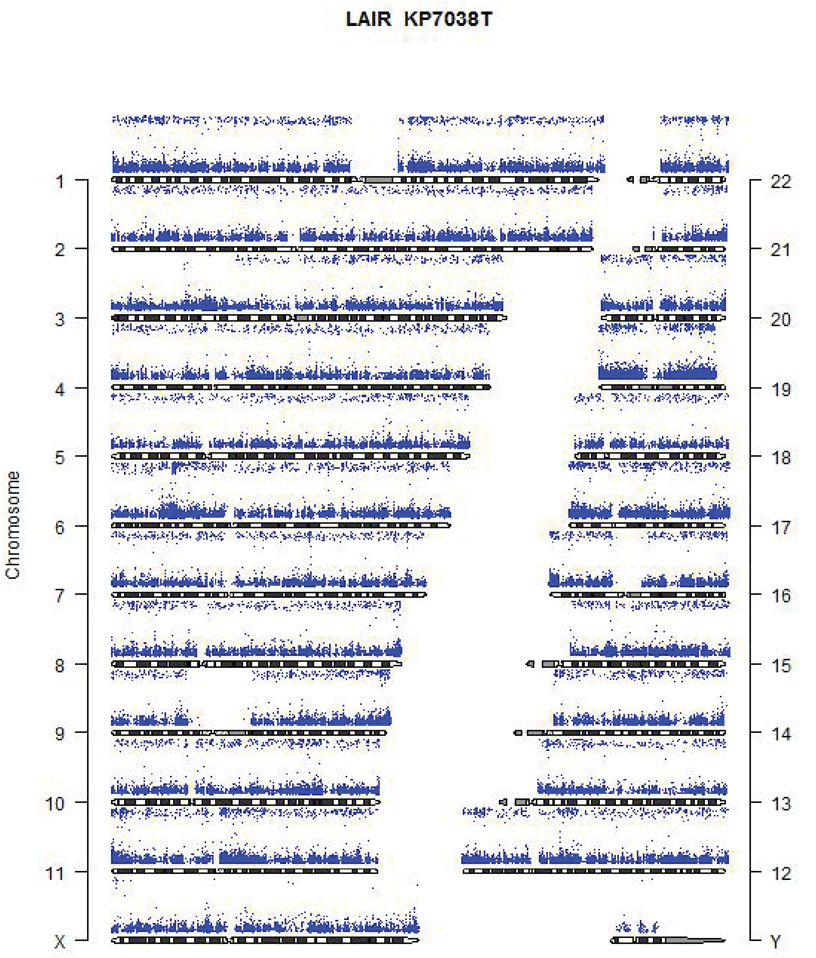
LAIR KP7038T
Chromosome
1
2
3
4
5
6
7
8
9
10
11
X
22
21
20
19
18
17
16
15
14
13
12
Y
